# Comparative Analysis of Foundational, Advanced, and Traditional Deep Learning Models for Hyperpolarized Gas MRI Lung Segmentation: Robust Performance in Data-Constrained Scenarios

**DOI:** 10.3390/bioengineering12101062

**Published:** 2025-09-30

**Authors:** Ramtin Babaeipour, Matthew S. Fox, Grace Parraga, Alexei Ouriadov

**Affiliations:** 1 School of Biomedical Engineering, Faculty of Engineering, The University of Western Ontario, London, ON N6A 3K7, Canada; rbabaeip@uwo.ca (R.B.);; 2 Department of Physics and Astronomy, The University of Western Ontario, London, ON N6A 3K7, Canada; 3 Lawson Health Research Institute, London, ON N6C 2R5, Canada; 4 Department of Medical Biophysics, The University of Western Ontario, London, ON N6A 3K7, Canada; 5 Robarts Research Institute, London, ON N6A 5B7, Canada

**Keywords:** foundational models, hyperpolarized gas MRI, limited data, medical imaging, segmentation

## Abstract

This study investigates the comparative performance of foundational models, advanced large-kernel architectures, and traditional deep learning approaches for hyperpolarized gas MRI segmentation across progressive data reduction scenarios. Chronic obstructive pulmonary disease (COPD) remains a leading global health concern, and advanced imaging techniques are crucial for its diagnosis and management. Hyperpolarized gas MRI, utilizing helium-3 (^3^He) and xenon-129 (^129^Xe), offers a non-invasive way to assess lung function. We evaluated foundational models (Segment Anything Model and MedSAM), advanced architectures (UniRepLKNet and TransXNet), and traditional deep learning models (UNet with VGG19 backbone, Feature Pyramid Network with MIT-B5 backbone, and DeepLabV3 with ResNet152 backbone) using four data availability scenarios: 100%, 50%, 25%, and 10% of the full training dataset (1640 2D MRI slices from 205 participants). The results demonstrate that foundational and advanced models achieve statistically equivalent performance across all data scenarios (*p* > 0.01), while both significantly outperform traditional architectures under data constraints (*p* < 0.001). Under extreme data scarcity (10% training data), foundational and advanced models maintained DSC values above 0.86, while traditional models experienced catastrophic performance collapse. This work highlights the critical advantage of architectures with large effective receptive fields in medical imaging applications where data collection is challenging, demonstrating their potential to democratize advanced medical imaging analysis in resource-limited settings.

## 1. Introduction

The global burden of chronic respiratory diseases continues to rise. Chronic obstructive pulmonary disease (COPD) is the third leading cause of death worldwide, accounting for 3.23 million deaths in 2019 [1].

Imaging modalities such as chest X-rays, CT scans, MRI, and Nuclear Medicine enable accurate diagnosis of the severity and type of COPD, ensuring appropriate treatment to alleviate symptoms and enhance daily functioning. Advanced imaging techniques can track the progression of COPD and identify complications, allowing healthcare providers to forecast potential outcomes and adjust treatment plans to improve prognosis and quality of life.

Chest CT is considered the current diagnostic tool for assessing structural abnormalities in the lungs, particularly in COPD patients [2]. It is regarded as the clinical gold standard for pulmonary imaging due to its outstanding spatial and temporal resolution. Chest CT findings correlate strongly with pathological results from post-mortem studies, confirming the presence and extent of emphysema [3]. However, despite its diagnostic advantages, CT significantly contributes to medical radiation exposure, raising concerns about the risk of radiation-induced cancer [4]. This is especially worrisome given the increasing frequency of CT scans for longitudinal studies. Therefore, cautious use of CT imaging and consideration of alternative imaging methods are important to mitigate these risks.

Magnetic Resonance Imaging (MRI) is highly valued for its ability to perform repeated studies without exposing patients to ionizing radiation. MRI provides both structural and functional information, enabling comprehensive lung assessments, including airway structures, lung parenchyma, and blood flow [5]. Although traditionally underutilized for lung imaging due to challenges like low proton density and motion artifacts, recent advancements in MRI technology, such as faster imaging techniques and lung-specific contrast agents, have greatly enhanced its effectiveness and reliability.

Hyperpolarized gas MRI, using helium-3 (^3^He) and xenon-129 (^129^Xe), has revolutionized pulmonary imaging over the past three decades [6]. This technique offers detailed structural and functional insights into the lungs. Pioneered by Albert and colleagues in 1994, the polarization of ^129^Xe (^3^He) gas can be significantly increased through spin-exchange with optically pumped rubidium vapor, amplifying the MRI signal by approximately 100,000 times [7]. Hyperpolarized ^3^He and ^129^Xe MRI provide unparalleled advantages, including detailed visualization of airway structures, lung ventilation, and gas exchange processes [8]. Extensive research has confirmed the safety and tolerability of these gases, supporting their use in diverse research and clinical settings to provide critical information on lung function and disease.

Hyperpolarized gas MRI enables the quantification of specific biomarkers, offering detailed insights into lung health. One key biomarker, the Ventilation Defect Percent (VDP), measures the percentage of lung volume that is not effectively ventilated. This is visualized by areas lacking inhaled hyperpolarized gas on MRI, indicating poor ventilation. VDP is essential for assessing functional impairment in diseases like COPD [9].

Earlier studies for hyperpolarized gas MRI ventilation segmentation employed classical image processing and machine learning approaches [10], such as hierarchical K-means [11] and spatial fuzzy c-means (SFCM) clustering [12]; however, these algorithms are time consuming and observer dependent. For example, segmentation of a 16 slice MRI would take around 45 min which may not be applicable in a clinical setting.

Deep learning (DL) has shown significant promise in image segmentation [13]. The application of DL in medical imaging has advanced, providing accurate and efficient analysis of medical images. DL-based segmentation offers precise quantitative analysis and standardized indicators for clinical trials. For instance, the U-Net architecture [14], combined with the pre-trained DenseNet121, has been used for segmenting lung CT scans to detect COVID-19 abnormalities [15]. Additionally, Sulaiman et al. detailed a CNN architecture for segmenting lung diseases using chest X-ray images in Diagnostics [16].

DL applications in hyperpolarized gas MRI segmentation have also been explored. Studies include a large-scale investigation of ventilated lung segmentation using multi-nuclear hyperpolarized gas MRI [17], a dual-channel DL approach for lung cavity estimation from hyperpolarized gas and proton MRI [18], a 3D CNN-based method for ventilated lung segmentation [19], and a U-Net++ based Quantification of Ventilation Defects for ventilated lung segmentation [20]. These studies primarily used CNN methods for segmentation tasks.

Despite the previous research on DL-based segmentation of hyperpolarized gas MRI, significant gaps remain in understanding how different architectural paradigms perform under data-constrained conditions. A recent review identified a notable gap in the use of vision transformers and foundational models for this task [21]. A recent study [22] compared CNNs and Vision Transformer-based models for lung segmentation in proton and hyperpolarized gas MRI under varying noise levels, finding that transformer-based architectures such as SegFormer outperform CNNs in high-noise settings, underscoring their potential for robust clinical deployment. Recent developments have introduced foundational models pre-trained on vast datasets and advanced large-kernel architectures that challenge conventional design principles. The limited comparative analysis of these emerging architectural approaches for hyperpolarized gas MRI presents a clear opportunity to explore how different paradigms could enhance segmentation accuracy and efficiency, particularly when working with limited datasets.

The broader computer vision field has witnessed the emergence of three distinct architectural paradigms with different approaches to achieving robust performance. Traditional deep learning architectures, such as U-Net [23], Feature Pyramid Networks (FPNs) [24], and DeepLabV3 [25], rely on increasing network depth and complexity to capture spatial relationships, but typically require large datasets to achieve optimal performance. Foundational models, like the Segment Anything Model (SAM) [26] and its medical variant MedSAM [27], demonstrate remarkable performance through pre-training on vast, diverse datasets, offering potential advantages in data-scarce scenarios through transfer learning capabilities. Advanced large-kernel architectures, such as UniRepLKNet [28] and TransXNet [29], have shown that innovative kernel designs and hybrid attention mechanisms can achieve superior spatial context capture without requiring extensive pre-training datasets.

Recent developments in computer vision have continued to push architectural boundaries through innovative approaches to spatial context modeling and feature interaction. OverLoCK [30] demonstrates that pure ConvNets can effectively incorporate top-down attention through Deep-stage Decomposition Strategy (DDS) and Context-Mixing Dynamic Convolution (ContMix), achieving superior accuracy-efficiency trade-offs compared to ConvNeXt while maintaining ConvNet inductive biases. The emergence of non-causal Vision Mamba architectures, exemplified by VSSD [31], addresses fundamental limitations of causal state space models in vision tasks by preserving relative interaction weights while enabling bidirectional context modeling. Multi-scale approaches have gained prominence through MSVMamba [32], which introduces hierarchy-in-hierarchy designs with economical multi-scale scanning for efficient long-range dependency captureand SparX-Mamba [33], which enhances both Vision Mamba and transformer architectures through sparse cross-layer connections that improve feature reuse across distant layers with minimal computational overhead. These advances underscore the ongoing architectural innovation in computer vision, where diverse approaches, from enhanced ConvNets to non-causal state space models, continue to explore optimal strategies for spatial context aggregation and feature interaction, complementing the foundational model and large-kernel architecture paradigms examined in our study.

A critical challenge in medical imaging, particularly in specialized modalities like hyperpolarized gas MRI, is the scarcity of annotated data. Medical image acquisition is expensive, time-consuming, and often requires specialized equipment and expertise. Patient recruitment can be challenging due to ethical considerations, rare disease prevalence, and geographical limitations. Expert annotation is labor-intensive and requires specialized knowledge, creating bottlenecks in dataset creation. These constraints are particularly pronounced in hyperpolarized gas MRI, where the technology is available in limited centers worldwide and requires specialized expertise for both acquisition and interpretation.

Traditional deep learning models typically require large datasets to achieve optimal performance, making them vulnerable to overfitting and poor generalization when trained on limited data. This limitation poses significant challenges for clinical translation and widespread adoption of automated segmentation tools in specialized imaging modalities. However, foundational models are trained on vast, diverse datasets and learn generalizable representations that can be fine-tuned for specific applications. Similarly, advanced large-kernel architectures incorporate design principles that enhance spatial context capture and feature reuse efficiency. Both approaches theoretically offer several advantages in medical imaging scenarios: reduced data requirements for achieving good performance, improved generalization across different imaging conditions and populations, faster convergence during fine-tuning, and potential for robust performance under data constraints.

Despite these architectural advances and the recognized challenges of data scarcity in medical imaging, a critical gap remains in understanding the comparative performance of different architectural paradigms under systematically varied data constraints. While individual studies have explored foundational models, advanced architectures, or traditional approaches in isolation, there lacks a comprehensive comparative framework that evaluates these paradigms across identical datasets and progressive data reduction scenarios. This gap is particularly pronounced in specialized imaging modalities like hyperpolarized gas MRI, where practitioners face uncertainty about optimal architectural choices when working with inherently limited datasets. Furthermore, the absence of systematic evidence comparing the data efficiency of foundational pre-training approaches versus innovative architectural designs leaves clinicians and researchers without clear guidance for model selection in resource-constrained environments. Without this comparative understanding, the medical imaging community cannot make informed decisions about resource allocation, computational requirements, and expected performance trade-offs when implementing automated segmentation tools in clinical practice.

This study addresses the critical question of whether foundational models and advanced architectures truly offer advantages over traditional deep learning architectures when working with limited medical imaging data. Our research aims to provide a comprehensive comparative analysis between foundational models (SAM and MedSAM), advanced large-kernel architectures (UniRepLKNet and TransXNet), and established traditional architectures (UNet with VGG19, FPN with MIT-B5, and DeepLabV3 with ResNet152) for both proton MRI and hyperpolarized gas MRI segmentation.

Our specific contributions are as follows: (1) A systematic comparison of foundational, advanced, and traditional models using identical datasets and evaluation metrics. (2) Assessment of model performance across progressive data reduction scenarios (100%, 50%, 25%, and 10% of the original dataset).

This research provides crucial insights for the medical imaging community regarding the practical benefits of foundational and advanced models in real-world clinical scenarios where data availability is inherently limited, ultimately informing decisions about model selection and resource allocation for medical AI development.

## 2. Materials and Methods

This section describes the proton and hyperpolarized gas MRI dataset compilation, ground truth generation procedures, and deep learning architectures employed in this comparative analysis. All computational experiments were conducted using the PyTorch 2.0 framework on dual NVIDIA GeForce RTX 3090 GPUs (NVIDIA Corporation, Santa Clara, CA, USA).

### 2.1. Image Acquisition and Dataset Compilation

The imaging dataset utilized in this study was retrospectively compiled from multiple research and clinical investigations involving patients referred for hyperpolarized gas MRI scans. This study was performed following institutional ethics approval, with informed written consent obtained from all participants. Each of the scans in the dataset was accompanied by a semi-automated expert segmentation, serving as the ground truth.

Imaging data were acquired using a 3T Discovery MR750 scanner (GE Healthcare, Waukesha, WI, USA) equipped with flexible vest quadrature coils (Clinical MR Solutions, Milwaukee, WI, USA). Hyperpolarized ^129^Xe gas was prepared using a 9820 polarizer (Polarean, Durham, NC, USA) achieving polarization levels between 10–40%.

Proton MRI acquisition employed a fast-spoiled gradient-recalled echo sequence with the following parameters: TR/TE/flip angle = 4.7 ms/1.2 ms/30°; field-of-view = 40 × 40 cm^2^; bandwidth = 24.4 kHz; acquisition matrix = 128 × 80, zero-padded to 128 × 128; partial-echo = 62.5%; slice thickness = 15 mm with 15–17 slices. Hyperpolarized ^129^Xe imaging utilized a three-dimensional FGRE sequence (TR/TE = 6.7 ms/1.5 ms; variable flip-angle; field-of-view = 40 × 40 cm^2^; bandwidth = 15.63 kHz; matrix = 128 × 128; slice thickness = 15 mm with 14 slices).

Standardized breathing protocols ensured consistent lung volumes across modalities. Participants were trained to inhale 1.0 L of gas mixture from functional residual capacity: nitrogen for proton imaging and a combination of 400 mL hyperpolarized ^129^Xe with 600 mL ^4^He for gas imaging.

### 2.2. Ground Truth Generation and Quality Control

Ground truth lung segmentation masks were generated using a semi-automated pipeline implemented in MATLAB R2021b (The MathWorks, Natick, MA, USA). The process began with automated thresholding of hyperpolarized ^129^Xe gas images, followed by manual adjustment to exclude tracheal regions from the segmentation mask. Image registration between proton and hyperpolarized gas images required manual placement of 3–6 anatomical landmarks on both ^1^H and ^129^Xe images, with subsequent affine transformation calculation for spatial alignment. Thoracic cavity segmentation was obtained through region growing algorithms applied to the registered gas mask. All semi-automated segmentation results underwent comprehensive quality control review by five independent observers with varying levels of experience in hyperpolarized gas MRI analysis (range: 1–7 years of experience).

### 2.3. Dataset Characteristics and Stratification

We collected data from 205 participants, which included 22 healthy individuals, 26 with COPD, 90 with asthma, and 67 with Long-COVID-19. The study population had a mean age of 54 ± 16 years, included 119 females (58%), and had a mean BMI of 28 ± 6 kg/m^2^. This yielded 1640 2D slices, originally sized 128 × 128. Proton and hyperpolarized slices were registered using a CNN-based registration approach as described by Mozaffaripour et al. [34]. The dataset was balanced to ensure an even distribution across the participant groups, with an 80% training, 10% validation, and 10% testing split. To avoid data leakage, no specific patient data were included in both the training and testing sets. To ensure representativeness and fairness, we also maintained an even distribution of the different conditions across the training, validation, and test sets.

To ensure rigorous and unbiased model comparison, identical dataset splits were maintained across all architectural evaluations. The stratified splitting methodology addressed two critical distribution considerations: anatomical slice representation and disease condition balance. For anatomical representativeness, slices were categorized by their position within each participant’s imaging volume (apical, middle, and basal lung regions) and distributed proportionally across training, validation, and test sets to ensure representative coverage of complete lung anatomy.

Simultaneously, 205 participants were stratified by clinical condition with proportional representation maintained within each data split, ensuring that training (80%), validation (10%), and test (10%) sets each contained the same relative distribution of disease conditions. This dual-stratification approach prevented potential confounding effects from anatomical bias or disease-specific learning advantages, while consistent use of identical splits across all model architectures eliminated inter-model variability due to data distribution differences.

### 2.4. Preprocessing

Preprocessing was conducted using the MONAI library [35]. The intensity values of the images were normalized to a range of 0 to 255, while label intensities were scaled between 0 and 1, aligning with the expected input ranges for subsequent processing steps. Spatial padding was applied to standardize dimensions across all images and labels. Further preprocessing involved converting grayscale images to RGB format by replicating grayscale values across three channels. Ground truth masks were adjusted to have positive values for labels and zero for the background. Finally, bounding boxes to the full size of the image for each mask were determined to serve as prompts for the SAM and MedSAM processors.

These preprocessing steps were selected to ensure compatibility across diverse model architectures while preserving medical image characteristics. Intensity normalization to 0–255 range standardized input distributions across different MRI acquisition parameters and enabled consistent model convergence. Grayscale-to-RGB conversion was necessary because all evaluated models were pre-trained on natural RGB images (ImageNet) and required three-channel inputs; replicating grayscale values across channels preserved the original intensity information while maintaining compatibility with pre-trained feature extractors. Spatial padding standardized dimensions to prevent artifacts from variable image sizes, while full-image bounding boxes for foundational models ensured fair comparison by eliminating spatial guidance advantages that could confound architectural performance differences.

The choice of full-image bounding boxes as prompts for SAM and MedSAM was deliberate to ensure fair comparison with advanced and traditional models. Unlike foundational models that utilize prompts to guide segmentation, advanced architectures and traditional models process the entire image without any spatial guidance or region-of-interest indicators. By providing bounding boxes encompassing the full image dimensions rather than tight boxes around lung regions, we eliminated the potential advantage that precise spatial prompts could provide to foundational models, ensuring that all architectures operated under equivalent conditions where they must identify and segment lung structures from the complete image without prior localization information. This methodological approach prevents confounding effects that could arise from differential spatial guidance across model types and maintains the integrity of our comparative analysis focused on architectural resilience rather than prompt engineering advantages.

### 2.5. Model Architectures and Fine-Tuning

#### 2.5.1. Foundational Models

Foundation models are expansive AI systems trained on vast datasets using substantial computational resources, capable of generating diverse outputs from text to images. Prominent examples include Open AI’s GPT model [36], DALLE-3 [37], Segment Anything [26], and BERT [38].

The SAM, developed by Meta AI, is a foundational model for image segmentation. It features a revolutionary architecture consisting of three main components: the image encoder, prompt encoder, and mask decoder. The image encoder, using a Masked Autoencoder (MAE) with a Vision Transformer, creates an embedding by extracting essential features from the input image. This embedding is a 16x downscaled version of the original image, optimizing for efficient processing while retaining critical features.

Various types of prompts, including points, boxes, masks, and text, can be used with SAM. For our task, we employed box prompts. Each box is represented by an embedding pair, combining the positional encoding of its top-left and bottom-right corners with learned embeddings representing these positions. The lightweight mask decoder, a modified Transformer decoder block followed by a dynamic mask prediction head, predicts segmentation masks by integrating information from the image and prompt embeddings.

Despite their capabilities, foundational models like SAM have limited applicability in medical image segmentation due to the significant differences between natural and medical images. To address these challenges, MedSAM was developed as an extension of SAM, specifically tailored for the medical imaging domain. MedSAM is trained on a large-scale dataset comprising over one million medical image-mask pairs.

#### 2.5.2. Advanced Large-Kernel Architectures

UniRepLKNet [28], employs very large convolution kernels (up to 13 × 13) with four architectural guidelines: (1) use efficient structures to increase depth, (2) use dilated re-parameterization blocks to enhance large kernel performance, (3) decide kernel size by downstream task with large kernels typically used in middle and high-level layers, and (4) add small kernels when scaling up model depth. The core principle is that large kernels can “see wide without going deep,” enabling efficient spatial context capture. We evaluated UniRepLKNet-F (6.2M parameters) and UniRepLKNet-S (55.6M parameters) variants. We also employed TransXNet [29], A hybrid CNN-transformer architecture featuring a Dual Dynamic Token Mixer (D-Mixer) that simultaneously learns global and local dynamics. The D-Mixer combines overlapping spatial reduction attention (OSRA) for global context with input-dependent depthwise convolution (IDConv) for dynamic local feature extraction. The architecture also incorporates multiscale feedforward networks (MS-FFN) for comprehensive feature aggregation. We assessed TransXNet-tiny (12.8M parameters), TransXNet-small (26.9M parameters), and TransXNet-base (29.0M parameters).

#### 2.5.3. Traditional Deep Learning Models

As for the traditional deep learning models, the U-Net [14] architecture with VGG19 [39] encoder represents a well-established approach for medical image segmentation. VGG19 provides a robust feature extraction backbone with its deep convolutional layers, while the U-Net decoder enables precise spatial localization through skip connections. This combination has proven effective across numerous medical imaging applications.

FPN [24] architecture excels at multi-scale feature extraction, crucial for segmenting structures of varying sizes in medical images. The MIT-B5 [40] backbone, part of the Mix Transformer family, incorporates attention mechanisms and hierarchical feature learning, providing strong representational capabilities for complex segmentation tasks.

DeepLabV3 [41] employs atrous-convolution and pyramid pooling to capture multi-scale contextual information. The ResNet152 [42] backbone provides deep feature extraction capabilities with residual connections, enabling training of very deep networks while maintaining gradient flow and avoiding degradation problems.

### 2.6. Training Methodology

During fine-tuning, images and their corresponding bounding box prompts were processed and fed into the model. We employed the Adam optimizer [43] with learning rates carefully tuned for each model based on preliminary experiments. A combined Dice and Cross-Entropy loss function was used to optimize both overlap and boundary accuracy.

Early stopping based on validation loss prevented overfitting, ensuring model robustness and generalization capabilities. Detailed training specifications including hyperparameters, optimization strategies, and reproducibility specifications are provided in the Supplementary Materials.

### 2.7. Computational Complexity of Experimented Models

The evaluated models demonstrated significant variation in computational requirements and parameter counts, which have important implications for clinical deployment scenarios. Foundational models required the highest computational resources, with both SAM and MedSAM utilizing ViT-B backbones containing approximately 91 million parameters each. Among advanced architectures, UniRepLKNet variants showed considerable scalability, ranging from UniRepLKNet-F with 6.2 million parameters to UniRepLKNet-S with 55.6 million parameters. TransXNet architectures provided a middle-ground approach with TransXNet-tiny (12.8 M parameters), TransXNet-small (26.9 M parameters), and TransXNet-base (48.0 M parameters). Traditional deep learning models exhibited diverse parameter counts: UNet-VGG19 was the most lightweight at 34.5 million parameters, DeepLabV3-ResNet152 contained 60.1 million parameters, while FPN-MIT-B5 required 81.4 million parameters. Table 1 summarizes the parameter counts.

### 2.8. Progressive Data Reduction Experiments

To provide a comprehensive understanding of architectural resilience to data scarcity, we conducted experiments across four different training data availability scenarios while maintaining consistent validation and testing sets to enable direct performance comparison:

- 100% Training Data (Baseline): All models were trained on the complete training dataset (80% of total data) to establish optimal performance benchmarks under ideal conditions.
- 50% Training Data: Models were trained using half of the original training data, randomly sampled while maintaining class distribution balance, to assess moderate data reduction impact.
- 25% Training Data: Models were trained on a quarter of the original training data to simulate realistic data-constrained scenarios commonly encountered in specialized imaging modalities.
- 10% Training Data: Models were trained using only one-tenth of the original training data to represent extreme data scarcity conditions and test architectural robustness limits.

For the progressive data reduction experiments (50%, 25%, and 10% training data scenarios), the same dual-stratification methodology was applied when subsampling from the original training set, ensuring that each reduced training subset maintained proportional representation of both anatomical slice positions and disease conditions, thereby preserving dataset balance across all experimental conditions. Additionally, the same validation and testing sets were maintained to enable direct performance comparison across data availability conditions. Each model was trained until convergence with early stopping based on validation loss to prevent overfitting.

**Figure 1:**
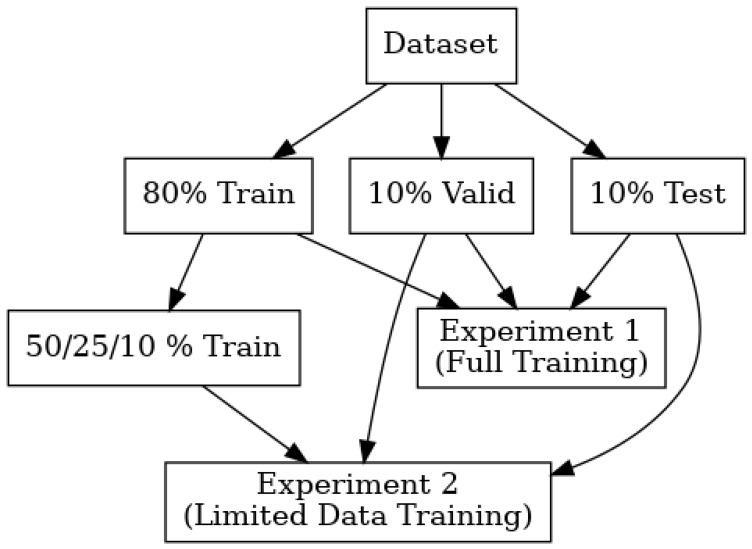
Experimental design flowchart illustrating the comparative analysis framework. The complete dataset was divided into 80% training, 10% validation, and 10% testing sets. Four progressive data reduction experiments were conducted using 100%, 50%, 25%, and 10% of the original training data while maintaining identical validation and testing sets across all scenarios to enable direct performance comparison between different data availability conditions.

A graph representing the experiments is depicted in Figure 1.

### 2.9. Evaluation Metrics and Statistical Analysis

Model performance was assessed using multiple complementary metrics to provide a comprehensive evaluation of segmentation quality. Primary metrics included Dice Similarity Coefficient (DSC) for overlap assessment, Hausdorff Distance at 95th percentile (HD95) for boundary accuracy, Average Hausdorff Distance (Avg HD) for overall geometric agreement, and XOR Error for pixel-wise disagreement quantification.

Statistical analysis employed IBM SPSS version 31 software with non-parametric tests due to the non-normal distribution of metrics as determined by Shapiro–Wilk tests. Friedman tests assessed differences between models, with post hoc pairwise comparisons using Bonferroni correction for multiple comparison adjustment. The analysis specifically evaluated: (1) performance equivalence between foundational and advanced models, (2) performance differences between foundational/advanced models versus traditional approaches, and (3) statistical significance of performance changes across data reduction scenarios for each architectural group.

## 3. Results

### 3.1. Full Dataset Performance (100% Training Data)

#### 3.1.1. Proton MRI Results

Under optimal data availability, performance differences between architectural paradigms were minimal. Statistical analysis revealed no significant differences (*p* > 0.01) between any model pairs across all evaluation metrics, indicating equivalent performance under full data conditions.

Foundational models demonstrated strong baseline performance with MedSAM achieving DSC of 0.91 (range: 0.73–0.97) and HD95 of 3.99, while SAM achieved DSC of 0.91 (range: 0.74–0.97) and HD95 of 4.18. Advanced large-kernel architectures showed comparable performance with UniRepLKNet-S (DSC: 0.90, HD95: 4.23) and TransXNet-base (DSC: 0.90, HD95: 4.26). Traditional architectures achieved similar performance levels: FPN-MIT-B5 (DSC: 0.91, HD95: 4.18), UNet-VGG19 (DSC: 0.91, HD95: 4.37), and DeepLabV3-ResNet152 (DSC: 0.90, HD95: 4.32). These performance metrics are comprehensively summarized in Table 2.

#### 3.1.2. Hyperpolarized Gas MRI Results

Consistent patterns emerged across imaging modalities, with all architectures demonstrating comparable performance and no significant differences between model groups. Foundational models: MedSAM (DSC: 0.89, HD95: 4.89), SAM (DSC: 0.88, HD95: 4.66). Advanced models: UniRepLKNet-S (DSC: 0.87, HD95: 5.69), TransXNet-base (DSC: 0.87, HD95: 5.60). Traditional approaches: FPN-MIT-B5 (DSC: 0.87, HD95: 5.34), UNet-VGG19 (DSC: 0.88, HD95: 5.47), DeepLabV3-ResNet152 (DSC: 0.86, HD95: 5.71). Complete performance metrics for all evaluated models on hyperpolarized gas MRI are presented in Table 3.

### 3.2. 50% Training Data Performance:

Initial performance divergence patterns emerged as data availability decreased. Foundational and advanced models maintained stability while traditional architectures showed increased performance variability and early signs of degradation.

#### 3.2.1. Proton MRI Results

Foundational models (MedSAM DSC: 0.90, SAM DSC: 0.90) and advanced models (UniRepLKNet-S DSC: 0.90, TransXNet-base DSC: 0.90) demonstrated minimal performance degradation. Traditional models showed moderate decline: FPN-MIT-B5 (DSC: 0.87), DeepLabV3-ResNet152 (DSC: 0.84), UNet-VGG19 (DSC: 0.89). Statistical analysis began revealing significant differences between traditional models and the foundational/advanced groups. Detailed performance metrics for the 50% training data scenario on proton MRI are summarized in Table 4.

#### 3.2.2. Hyperpolarized Gas MRI Results

Similar resilience patterns observed with foundational models (MedSAM DSC: 0.88, SAM DSC: 0.86), advanced models (UniRepLKNet-S DSC: 0.86, TransXNet-base DSC: 0.87), while traditional models exhibited greater performance variability and more pronounced degradation. The corresponding performance metrics for hyperpolarized gas MRI under 50% training data conditions are provided in Table 5.

### 3.3. 25% Training Data Performance:

This scenario revealed dramatic architectural differences in data efficiency. Statistical analysis demonstrated that both foundational and advanced models significantly outperformed traditional architectures (*p* < 0.001) while showing no significant differences between themselves (*p* > 0.01).

#### 3.3.1. Proton MRI Results

**Foundational Models:** Exceptional stability with MedSAM (DSC: 0.90) and SAM (DSC: 0.90)

**Advanced Models:** Comparable resilience with UniRepLKNet-S (DSC: 0.90) and TransXNet-base (DSC: 0.90)

**Traditional Models:** Severe performance collapse with FPN-MIT-B5 (DSC: 0.75), DeepLabV3-ResNet152 (DSC: 0.75), and catastrophic failure of UNet-VGG19 (DSC: 0.42)

Complete performance breakdown for the 25% training data scenario on proton MRI is detailed in Table 6.

#### 3.3.2. Hyperpolarized Gas MRI Results

**Foundational Models:** MedSAM (DSC: 0.87), SAM (DSC: 0.86)

**Advanced Models:** UniRepLKNet-S (DSC: 0.86), TransXNet-base (DSC: 0.86)

**Traditional Models:** Substantial degradation with FPN-MIT-B5 (DSC: 0.72), DeepLabV3-ResNet152 (DSC: 0.66), UNet-VGG19 (DSC: 0.81)

Performance metrics for hyperpolarized gas MRI under 25% training data conditions are presented in Table 7.

### 3.4. 10% Training Data Performance:

Under extreme data limitation, the architectural performance hierarchy became definitively established. Foundational and advanced models maintained good performance while traditional approaches experienced complete failure.

#### 3.4.1. Proton MRI Results

**Foundational Models:** Remarkable stability with MedSAM (DSC: 0.90) and SAM (DSC: 0.89)

**Advanced Models:** Near-equivalent performance with UniRepLKNet-S (DSC: 0.89) and TransXNet-base (DSC: 0.90)

**Traditional Models:** Complete failure with FPN-MIT-B5 (DSC: 0.66), DeepLabV3-ResNet152 (DSC: 0.61), UNet-VGG19 (DSC: 0.39)

The extreme data scarcity results for proton MRI are comprehensively documented in Table 8.

#### 3.4.2. Hyperpolarized Gas MRI Results

**Foundational Models:** MedSAM (DSC: 0.86), SAM (DSC: 0.84)

**Advanced Models:** UniRepLKNet-S (DSC: 0.86), TransXNet-base (DSC: 0.86)

**Traditional Models:** Severe degradation with FPN-MIT-B5 (DSC: 0.42), DeepLabV3-ResNet152 (DSC: 0.61), UNet-VGG19 (DSC: 0.67)

Corresponding extreme data limitation results for hyperpolarized gas MRI are summarized in Table 9.

### 3.5. Comprehensive Statistical Analysis

Statistical evaluation across all data scenarios revealed consistent and significant patterns:

**Full Data Scenario (100%):** No significant differences between any model architectures (*p* > 0.01), confirming equivalent performance under optimal conditions across all three architectural paradigms.

**Progressive Data Reduction Scenarios (50%, 25%, 10%):**

**Foundational vs. Advanced Models:** No significant differences (*p* > 0.01) across all data scenarios, demonstrating statistical equivalence

**Foundational Models vs. Traditional Models:** Significant superiority (*p* < 0.001) in all data-limited scenarios

**Advanced Models vs. Traditional Models:** Identical significance pattern (*p* < 0.001) as foundational models versus traditional approaches

**Shared Statistical Profile:** Both foundational and advanced models exhibited identical statistical superiority patterns over traditional architectures

This statistical analysis confirms that foundational and advanced large-kernel models are statistically equivalent in performance while both groups significantly outperform traditional approaches under data constraints.

Comprehensive boxplot analysis across all progressive data reduction scenarios is presented in Supplementary Materials (Figures S1–S32), systematically documenting performance metrics for both proton and hyperpolarized gas MRI across 100%, 50%, 25%, and 10% training data conditions.

Effect size analysis using Cohen’s d revealed clinically meaningful differences between architectural paradigms that intensified with progressive data reduction. Under full data conditions (100% training data), all pairwise comparisons yielded negligible effect sizes (d < 0.2), confirming architectural equivalence when sufficient training data is available. However, as training data decreased to 50% and below, large effect sizes (d > 1.0) consistently emerged between foundational/advanced models versus traditional architectures, with some comparisons reaching d > 2.0 under extreme data scarcity (10% training data)—representing differences exceeding two pooled standard deviations. Notably, foundational models and advanced architectures maintained negligible effect sizes (d < 0.2) relative to each other across all data scenarios, confirming their statistical equivalence despite different underlying design principles. The magnitude of these effect sizes indicates not merely statistical significance but clinically substantial differences in segmentation quality, with traditional architectures experiencing performance degradation of sufficient magnitude to render them unsuitable for clinical deployment under data-constrained conditions. Comprehensive effect size heatmaps for all architectural comparisons across progressive data reduction scenarios are provided in the Supplementary Materials.

## 4. Discussion

This study provides comprehensive evidence that architectural approaches incorporating large effective receptive fields, whether through extensive pre-training (foundational models) or innovative kernel designs (advanced architectures), offer fundamental advantages over traditional deep learning approaches when working with limited medical imaging data. The results establish clear architectural performance hierarchies that become increasingly pronounced under data-constrained conditions, providing crucial guidance for clinical AI deployment. Representative qualitative comparisons illustrating these performance differences across model architectures are provided in the Supplementary Materials (Figures S33 and S34).

### 4.1. Statistical Equivalence of Foundational and Advanced Models

The most significant finding of our comprehensive analysis is the statistical equivalence between foundational models and advanced large-kernel architectures across all data availability scenarios. This equivalence demonstrates that innovative architectural designs can achieve the data efficiency benefits traditionally associated with extensive pre-training approaches, offering multiple viable pathways for robust performance under data constraints.

The success of UniRepLKNet validates the architectural principle that large kernels enable models to “see wide without going deep.” The dilated re-parameterization blocks and strategic kernel size selection created efficient spatial context aggregation that maintained robust performance even with severely limited training data. This approach effectively decouples the traditional relationship between model depth and receptive field size, enabling more efficient parameter utilization under data constraints.

TransXNet’s dual dynamic token mixer demonstrated that hybrid approaches combining global attention mechanisms with input-dependent local feature extraction achieve performance parity with foundational models. The overlapping spatial reduction attention for global context, combined with dynamic convolution for local features, created a balanced architecture capable of maintaining both spatial detail preservation and global context understanding across progressive data reduction scenarios.

### 4.2. Progressive Data Reduction Performance Analysis

Under optimal data conditions, the performance convergence across all architectural approaches suggests that traditional models can achieve competitive results when sufficient training data is available. This convergence validates that the fundamental task of lung segmentation can be addressed through multiple architectural paradigms when data limitations are not a constraint.

The 25% training data scenario emerged as a critical threshold where architectural differences become clinically significant. At this point, both foundational and advanced models maintained near-optimal performance (DSC > 0.86 for both modalities), while traditional architectures experienced severe degradation. This threshold represents a realistic clinical scenario where specialized imaging modalities face moderate data constraints.

Under 10% training data conditions, the exceptional stability of both foundational and advanced models (maintaining DSC > 0.84) contrasts dramatically with the complete failure of traditional approaches. This resilience addresses a fundamental barrier to clinical AI translation in specialized imaging modalities, demonstrating that high-quality automated segmentation remains achievable even under extreme data limitations.

Effect size analysis using Cohen’s d with 95% confidence intervals provided quantitative evidence for the magnitude of architectural performance differences across progressive data reduction scenarios (Supplementary Materials Figure S35). Under full data conditions (100% training data), all pairwise comparisons yielded negligible effect sizes (d < 0.2) with overlapping confidence intervals, confirming architectural equivalence when sufficient training data is available. However, as training data decreased, large effect sizes (d > 1.0) consistently emerged between foundational/advanced models versus traditional architectures, with some comparisons reaching d > 2.0 under extreme data scarcity (10% training data), representing differences exceeding two pooled standard deviations. Most critically, foundational models and advanced architectures maintained negligible effect sizes (d < 0.2) relative to each other across all data scenarios, providing statistical confirmation of their equivalence despite different underlying design principles. The magnitude of these effect sizes indicates not merely statistical significance but clinically substantial differences in segmentation quality, with traditional architectures experiencing performance degradation of sufficient magnitude to render them unsuitable for clinical deployment under data-constrained conditions, while both foundational and advanced approaches demonstrated statistical equivalence in their superior data efficiency.

### 4.3. Modality-Specific Considerations

While hyperpolarized gas MRI’s specialized imaging characteristics initially minimized architectural advantages under full data conditions, the benefits of foundational and advanced models became increasingly apparent with data reduction. This pattern suggests that specialized imaging modalities, which inherently face data scarcity challenges, represent primary beneficiaries of advanced architectural approaches.

The consistent performance advantages observed across both imaging modalities support the hypothesis that architectural approaches enhancing effective receptive field capture provide fundamental benefits for medical image segmentation. Both foundational pre-training and large-kernel designs enable efficient spatial context aggregation that reduces dependence on extensive training examples.

### 4.4. Clinical Translation and Practical Implications

The demonstrated data efficiency of both foundational and advanced models provides multiple pathways for democratizing advanced medical imaging analysis. The ability to achieve clinically acceptable performance with 75–90% less training data enables deployment in resource-limited settings, specialized centers, or emerging imaging modalities where extensive data collection is impractical.

The statistical equivalence between foundational and advanced models provides practical flexibility for clinical implementation. Selection can be based on computational resources, deployment constraints, regulatory considerations, and specific application requirements while maintaining confidence in performance reliability under data-limited conditions.

The availability of multiple equivalent approaches (foundational vs. advanced) enables optimization based on secondary factors such as computational efficiency, memory requirements, inference speed, or customization capabilities, while ensuring robust performance across varying data availability scenarios.

### 4.5. Computational Efficiency and Clinical Deployment Trade-Offs

The computational analysis reveals a nuanced relationship between model complexity and performance efficiency that has significant implications for clinical deployment strategies. While foundational models (SAM and MedSAM at 91M parameters each) achieved superior data efficiency, their computational demands may limit deployment in resource-constrained environments. However, the statistical equivalence demonstrated by advanced architectures offers compelling alternatives with more favorable computational profiles. UniRepLKNet-F, with only 6.2M parameters, achieved performance parity with models containing 15× more parameters, representing exceptional parameter efficiency. Similarly, TransXNet variants provided scalable options ranging from 12.8M to 48.0M parameters while maintaining equivalent performance to much larger foundational models. This finding challenges the conventional assumption that superior performance necessarily requires larger models, suggesting that architectural innovation can achieve foundational model-level benefits with significantly reduced computational overhead. For clinical translation, this implies that healthcare institutions with limited computational resources need not sacrifice performance quality, as advanced architectures like UniRepLKNet-F offer a compelling middle ground between traditional models’ data vulnerability and foundational models’ computational demands. The 14-fold reduction in parameters from foundational to advanced models while maintaining statistical performance equivalence represents a critical breakthrough for democratizing advanced medical imaging AI, enabling deployment across diverse clinical environments from resource-limited settings to high-throughput imaging centers. This computational flexibility, combined with demonstrated resilience under data constraints, positions advanced architectures as potentially optimal choices for widespread clinical adoption.

### 4.6. Limitations

Several limitations warrant consideration in interpreting these results. First, our analysis focused on segmentation tasks within specific anatomical regions (lung segmentation). The generalizability of these findings to other anatomical structures, pathological conditions, or segmentation tasks requires further investigation.

Secondly, our study did not extensively explore computational efficiency and resource requirements. While foundational models demonstrated superior performance, their larger memory footprints and computational demands may present practical challenges in resource-constrained clinical environments.

Thirdly, validation of the models on real patient data in clinical practice has been limited. Our evaluation was conducted on retrospectively collected research datasets, and prospective clinical validation would be necessary to confirm the practical utility and safety of these approaches in real-world diagnostic workflows.

Additionally, our dataset originates from a single center and has not been validated across different MRI devices, scanner manufacturers, imaging protocols, or diverse patient populations spanning different ethnicities and demographic groups. This single-center limitation restricts the generalizability of our findings and highlights the need for multi-center validation studies to establish robustness across varied clinical environments and patient populations.

To address these limitations, future research should prioritize multi-center validation studies incorporating diverse scanner manufacturers, imaging protocols, and patient demographics to establish broader generalizability. Federated learning approaches could enable collaborative training across institutions while preserving patient privacy, while domain adaptation techniques could facilitate rapid model deployment to new clinical sites with minimal local data requirements. Expanding evaluation beyond lung segmentation to other anatomical structures and pathological conditions would demonstrate architectural robustness across medical imaging applications. Additionally, comprehensive computational efficiency analyses comparing inference times, memory requirements, and energy consumption would provide practical deployment guidance for resource-constrained clinical environments. Prospective clinical validation studies integrating these models into real diagnostic workflows would ultimately confirm their safety, utility, and impact on clinical decision-making and patient outcomes.

## 5. Conclusions

This comprehensive evaluation across progressive data reduction scenarios provides compelling evidence that architectural design fundamentally determines model resilience to data scarcity in medical imaging applications. Our systematic comparison of foundational models, advanced large-kernel architectures, and traditional deep learning approaches across four data availability scenarios reveals consistent patterns that have profound implications for clinical AI deployment.

Our results demonstrate that both foundational models (SAM and MedSAM) and advanced architectures (UniRepLKNet and TransXNet) maintain robust performance when transitioning from full to severely limited data scenarios, with minimal degradation in segmentation accuracy across both imaging modalities. Most remarkably, these architectures achieve statistically equivalent performance (*p* > 0.01) across all data conditions, demonstrating that innovative architectural designs can match the benefits of extensive pre-training. In stark contrast, traditional architectures (UNet-VGG19, FPN-MIT-B5, DeepLabV3-ResNet152) suffer catastrophic performance collapse under data constraints, with some models experiencing DSC decreases exceeding 50%.

Importantly, while specialized imaging characteristics (as observed in hyperpolarized gas and proton MRI) may initially level performance differences between all architectural approaches under full data conditions, both foundational and advanced models retain their resilience advantages when data becomes scarce. Under extreme data scarcity (10% training data), both architectural groups maintained acceptable performance (DSC > 0.86) while traditional models experienced complete failure. This suggests that the benefits of both foundational approaches and advanced architectural innovations extend beyond simple domain transfer to encompass fundamental learning efficiency advantages that persist across imaging modalities.

These findings have profound implications for the deployment of AI tools in medical imaging workflows. The statistical equivalence between foundational and advanced models provides healthcare institutions with flexible implementation options based on their specific computational resources, regulatory constraints, and deployment requirements while maintaining confidence in performance reliability. The superior data efficiency of both architectural approaches could significantly lower barriers to implementing automated segmentation in specialized imaging applications, resource-limited settings, and emerging clinical domains where large datasets are difficult to obtain.

For hyperpolarized gas MRI specifically, our results suggest that both foundational and advanced models could enable more widespread adoption of automated biomarker quantification tools, potentially improving the accessibility and standardization of this important but specialized imaging technique. The ability to achieve clinically viable performance with 75–90% less training data addresses fundamental barriers to clinical translation in specialized imaging modalities that are available in limited centers worldwide.

Our findings suggest that the medical imaging community should prioritize architectural approaches that enhance effective receptive field capture. These approaches can be achieved through extensive pre-training, innovative kernel designs, or hybrid attention mechanisms. This paradigm shift from traditional depth-focused architectures to spatial context-focused designs could accelerate the development and deployment of AI tools across diverse clinical applications while improving accessibility in resource-constrained environments.

As medical imaging continues to evolve with new technologies and clinical applications, the need for robust, data-efficient AI tools becomes increasingly critical. Our results provide strong evidence that both foundational models and advanced architectural innovations offer promising paths forward, particularly for addressing the data scarcity challenges that have historically limited the clinical translation of AI tools in specialized medical imaging domains.

The consistent demonstration that architectural innovation can achieve foundational model-level performance while potentially offering greater deployment flexibility opens new avenues for practical clinical AI implementation. This revelation that multiple equivalent pathways exist for achieving robust performance under data-limited conditions ultimately advances the goal of democratizing advanced medical imaging analysis across diverse healthcare settings and improving patient care through more accessible automated diagnostic tools.

Building on these findings, our future research will pursue several interconnected avenues that leverage the demonstrated advantages of foundational and advanced architectural approaches in data-constrained medical imaging scenarios.

We plan to advance multimodal approaches that integrate language and vision capabilities for enhanced medical image segmentation. This involves developing hybrid architectures that can simultaneously process visual information alongside natural language descriptions. These descriptions will include anatomical structures, pathological findings, and clinical context. Specifically, we will explore how radiological reports, clinical notes, and structured medical vocabularies can be incorporated as additional guidance signals during the segmentation process. This includes investigating prompt engineering techniques that allow clinicians to provide natural language descriptions of regions of interest, pathological characteristics, or diagnostic priorities that can dynamically influence segmentation boundaries and accuracy. We will also develop cross-modal attention mechanisms that enable the model to align textual descriptions with corresponding visual features, potentially improving segmentation precision in ambiguous or challenging cases.

We will explore autonomous quality assessment and correction mechanisms that can iteratively refine segmentation results through self-supervised learning approaches. This involves developing meta-learning frameworks where models can evaluate their own segmentation quality using uncertainty quantification, anatomical plausibility checks, and consistency validation across multiple imaging planes or temporal sequences. These agentic systems will incorporate active learning components that can identify cases requiring human expert review, suggest targeted corrections, and adapt their performance based on accumulated feedback from clinical workflows.

Given the single-center limitation identified in this study, we plan comprehensive multi-center validation studies across different scanner manufacturers, imaging protocols, and patient populations to establish the generalizability of our architectural findings. This will include developing domain adaptation techniques that can rapidly fine-tune models for new clinical sites with minimal local data requirements, investigating federated learning approaches that can benefit from distributed clinical datasets while preserving patient privacy, and establishing standardized evaluation protocols for assessing model performance across diverse clinical environments.

This research directions aim to translate the fundamental architectural insights demonstrated in this study into practical, clinically deployable systems that can improve patient care while addressing the inherent data scarcity challenges in specialized medical imaging domains.

## Figures and Tables

**Table 1:** Approximate parameter counts of the selected segmentation models, highlighting their relative computational complexity.

Model	Parameters (Approx.)
**SAM ViT-B**	~91 M
**MedSAM ViT-B**	~91 M
**UniRepLKNet-F**	~6.2 M
**UniRepLKNet-S**	~55.6 M
**FPN_mit_b5**	~81.4 M
**DeepLabV3_resnet152**	~60.1 M
**Unet_vgg19**	~34.5 M
**TransXNet_tiny**	~12.8 M
**TransXNet_small**	~26.9 M
**TransXNet_base**	~48.0 M

**Table 2:** Performance metrics for all model architectures on the proton MRI full dataset experiment (100% training data). Values are presented as mean for Dice Similarity Coefficient (DSC), Average Hausdorff Distance (Avg_HD), 95th percentile Hausdorff Distance (HD95), and XOR error.

Model	DSC	Avg_HD	HD95	XOR
**DeepLabV3_resnet152**	0.90	1.35	4.32	0.18
**FPN_mit_b5**	0.91	1.34	4.18	0.17
**MedSAM**	0.91	1.31	3.99	0.17
**SAM**	0.91	1.35	4.18	0.17
**TransXNet_base**	0.90	1.34	4.26	0.17
**TransXNet_small**	0.90	1.34	4.19	0.18
**TransXNet_tiny**	0.90	1.29	4.26	0.17
**Unet_vgg19**	0.91	1.32	4.37	0.17
**UniRepLKNet-F**	0.91	1.28	4.06	0.16
**UniRepLKNet-S**	0.90	1.25	4.23	0.17

**Table 3:** Performance metrics for all model architectures on the hyperpolarized gas MRI full dataset experiment (100% training data). Values are presented as mean for Dice Similarity Coefficient (DSC), Average Hausdorff Distance (Avg_HD), 95th percentile Hausdorff Distance (HD95), and XOR error.

Model	DSC	Avg_HD	HD95	XOR
**DeepLabV3_resnet152**	0.86	1.58	5.71	0.26
**FPN_mit_b5**	0.87	1.65	5.34	0.27
**MedSAM**	0.89	1.46	4.89	0.23
**SAM**	0.88	1.43	4.66	0.22
**TransXNet_base**	0.87	1.50	5.60	0.25
**TransXNet_small**	0.87	1.57	5.42	0.25
**TransXNet_tiny**	0.87	1.64	5.43	0.26
**Unet_vgg19**	0.88	1.52	5.47	0.25
**UniRepLKNet-F**	0.87	1.65	5.76	0.27
**UniRepLKNet-S**	0.87	1.58	5.69	0.26

**Table 4:** Performance metrics for all model architectures on the proton MRI limited dataset experiment (50% training data). Values are presented as mean for Dice Similarity Coefficient (DSC), Average Hausdorff Distance (Avg_HD), 95th percentile Hausdorff Distance (HD95), and XOR error.

Model	DSC	Avg_HD	HD95	XOR
**DeepLabV3_resnet152**	0.84	2.12	7.28	0.30
**FPN_mit_b5**	0.87	1.76	5.79	0.25
**MedSAM**	0.90	1.37	4.21	0.18
**SAM**	0.90	1.41	4.38	0.18
**TransXNet_base**	0.90	1.34	4.67	0.18
**TransXNet_small**	0.90	1.45	4.49	0.19
**TransXNet_tiny**	0.90	1.39	4.66	0.19
**Unet_vgg19**	0.89	1.89	5.76	0.21
**UniRepLKNet-F**	0.90	1.39	4.40	0.18
**UniRepLKNet-S**	0.90	1.41	4.34	0.18

**Table 5:** Performance metrics for all model architectures on xenon MRI limited dataset experiment (50% training data). Values are presented as mean for Dice Similarity Coefficient (DSC), Average Hausdorff Distance (Avg_HD), 95th percentile Hausdorff Distance (HD95), and XOR error.

Model	DSC	Avg_HD	HD95	XOR
**DeepLabV3_resnet152**	0.77	2.93	9.72	0.51
**FPN_mit_b5**	0.77	2.16	9.23	0.37
**MedSAM**	0.88	1.61	5.34	0.26
**SAM**	0.86	1.54	6.34	0.25
**TransXNet_base**	0.87	1.62	5.30	0.25
**TransXNet_small**	0.87	1.64	5.62	0.26
**TransXNet_tiny**	0.86	1.58	5.87	0.26
**Unet_vgg19**	0.66	3.16	12.16	0.48
**UniRepLKNet-F**	0.87	1.67	5.75	0.26
**UniRepLKNet-S**	0.86	1.62	6.17	0.26

**Table 6:** Performance metrics for all model architectures on proton MRI limited dataset experiment (25% training data). Values are presented as mean for Dice Similarity Coefficient (DSC), Average Hausdorff Distance (Avg_HD), 95th percentile Hausdorff Distance (HD95), and XOR error.

Model	DSC	Avg_HD	HD95	XOR
**DeepLabV3_resnet152**	0.75	5.10	15.28	0.59
**FPN_mit_b5**	0.75	3.43	11.77	0.46
**MedSAM**	0.90	1.40	4.35	0.18
**SAM**	0.90	1.40	4.35	0.18
**TransXNet_base**	0.90	1.39	4.44	0.19
**TransXNet_small**	0.90	1.45	4.36	0.19
**TransXNet_tiny**	0.89	1.48	4.64	0.20
**Unet_vgg19**	0.42	5.88	21.40	0.71
**UniRepLKNet-F**	0.90	1.41	4.31	0.18
**UniRepLKNet-S**	0.90	1.47	4.48	0.19

**Table 7:** Performance metrics for all model architectures on xenon MRI limited dataset experiment (25% training data). Values are presented as mean for Dice Similarity Coefficient (DSC), Average Hausdorff Distance (Avg_HD), 95th percentile Hausdorff Distance (HD95), and XOR error.

Model	DSC	Avg_HD	HD95	XOR
**DeepLabV3_resnet152**	0.66	3.36	17.20	0.56
**FPN_mit_b5**	0.72	3.26	12.76	0.59
**MedSAM**	0.87	1.78	5.51	0.28
**SAM**	0.86	1.62	5.88	0.27
**TransXNet_base**	0.86	1.80	5.73	0.28
**TransXNet_small**	0.86	1.74	5.92	0.28
**TransXNet_tiny**	0.87	1.78	5.97	0.28
**Unet_vgg19**	0.81	3.07	8.95	0.49
**UniRepLKNet-F**	0.87	1.66	5.57	0.27
**UniRepLKNet-S**	0.86	1.69	6.30	0.28

**Table 8:** Performance metrics for all model architectures on proton MRI limited dataset experiment (10% training data). Values are presented as mean for Dice Similarity Coefficient (DSC), Average Hausdorff Distance (Avg_HD), 95th percentile Hausdorff Distance (HD95), and XOR error.

Model	DSC	Avg_HD	HD95	XOR
**DeepLabV3_resnet152**	0.61	5.68	17.49	0.98
**FPN_mit_b5**	0.66	6.07	22.98	1.03
**MedSAM**	0.90	1.67	5.06	0.20
**SAM**	0.89	1.61	5.10	0.21
**TransXNet_base**	0.90	1.47	4.58	0.20
**TransXNet_small**	0.89	1.61	4.97	0.20
**TransXNet_tiny**	0.89	1.56	4.70	0.20
**Unet_vgg19**	0.39	4.71	33.13	0.74
**UniRepLKNet-F**	0.89	1.62	4.94	0.20
**UniRepLKNet-S**	0.89	1.47	4.52	0.19

**Table 9:** Performance metrics for all model architectures on xenon MRI limited dataset experiment (10% training data). Values are presented as mean for Dice Similarity Coefficient (DSC), Average Hausdorff Distance (Avg_HD), 95th percentile Hausdorff Distance (HD95), and XOR error.

Model	DSC	Avg_HD	HD95	XOR
**DeepLabV3_resnet152**	0.61	7.16	16.48	1.40
**FPN_mit_b5**	0.42	4.01	36.94	0.77
**MedSAM**	0.86	1.71	5.50	0.27
**SAM**	0.84	2.10	7.04	0.35
**TransXNet_base**	0.86	1.98	6.44	0.30
**TransXNet_small**	0.86	2.10	6.26	0.32
**TransXNet_tiny**	0.84	1.80	6.90	0.30
**Unet_vgg19**	0.67	3.15	12.85	0.50
**UniRepLKNet-F**	0.85	1.89	5.93	0.30
**UniRepLKNet-S**	0.86	1.73	5.60	0.28

## Data Availability

The original contributions presented in the study are included in the article/Supplementary Materials; further inquiries can be directed to the corresponding author.

## Supplementary Materials

The following supporting information can be downloaded at: https://www.mdpi.com/article/10.3390/bioengineering12101062/s1, Figure S1: Proton MRI segmentation performance (DSC) with full training data across foundational, advanced, and traditional models; Figure S2: Proton MRI segmentation performance (HD95) with full training data across all model architectures; Figure S3: Proton MRI segmentation performance (Avg_HD) with full training data across all model architectures; Figure S4: Proton MRI segmentation performance (XOR) with full training data across all model architectures; Figure S5: Hyperpolarized gas MRI segmentation performance (DSC) with full training data across all model architectures; Figure S6: Hyperpolarized gas MRI segmentation performance (HD95) with full training data across all model architectures; Figure S7: Hyperpolarized gas MRI segmentation performance (Avg_HD) with full training data across all model architectures; Figure S8: Hyperpolarized gas MRI segmentation performance (XOR) with full training data across all model architectures; Figure S9: Proton MRI segmentation performance (DSC) with 50% training data; Figure S10: Proton MRI segmentation performance (HD95) with 50% training data; Figure S11: Proton MRI segmentation performance (Avg_HD) with 50% training data; Figure S12: Proton MRI segmentation performance (XOR) with 50% training data; Figure S13: Hyperpolarized gas MRI segmentation performance (DSC) with 50% training data; Figure S14: Hyperpolarized gas MRI segmentation performance (HD95) with 50% training data; Figure S15: Hyperpolarized gas MRI segmentation performance (Avg_HD) with 50% training data; Figure S16: Hyperpolarized gas MRI segmentation performance (XOR) with 50% training data; Figure S17: Proton MRI segmentation performance (DSC) with 25% training data; Figure S18: Proton MRI segmentation performance (HD95) with 25% training data; Figure S19: Proton MRI segmentation performance (Avg_HD) with 25% training data; Figure S20: Proton MRI segmentation performance (XOR) with 25% training data; Figure S21: Hyperpolarized gas MRI segmentation performance (DSC) with 25% training data; Figure S22: Hyperpolarized gas MRI segmentation performance (HD95) with 25% training data; Figure S23: Hyperpolarized gas MRI segmentation performance (Avg_HD) with 25% training data; Figure S24: Hyperpolarized gas MRI segmentation performance (XOR) with 25% training data; Figure S25: Proton MRI segmentation performance (DSC) with 10% training data; Figure S26: Proton MRI segmentation performance (HD95) with 10% training data; Figure S27: Proton MRI segmentation performance (Avg_HD) with 10% training data; Figure S28: Proton MRI segmentation performance (XOR) with 10% training data; Figure S29: Hyperpolarized gas MRI segmentation performance (DSC) with 10% training data; Figure S30: Hyperpolarized gas MRI segmentation performance (HD95) with 10% training data; Figure S31: Hyperpolarized gas MRI segmentation performance (Avg_HD) with 10% training data; Figure S32: Hyperpolarized gas MRI segmentation performance (XOR) with 10% training data; Figure S33: Qualitative comparison of hyperpolarized gas MRI lung segmentation across progressive data reduction scenarios; Figure S34: Qualitative comparison of proton MRI lung segmentation across progressive data reduction scenarios; Figure S35: Cohen’s d effect sizes with 95% confidence intervals comparing architectural paradigms across evaluation metrics.
