# Supplementary material for: Comparative Analysis of Foundational, Advanced, and Traditional Deep Learning Models for Hyperpolarized Gas MRI Lung Segmentation: Robust Performance in Data-Constrained Scenarios
Source: Bioengineering (Basel). 2025 Sep 30;12(10):1062. doi: 10.3390/bioengineering12101062 (PMC12561172; doi:10.3390/bioengineering12101062)
Supplement: Supplementary file 1 [file bioengineering-12-01062-s001.zip › bioengineering-3850026-supplementary.pdf]

Supplementary Materials

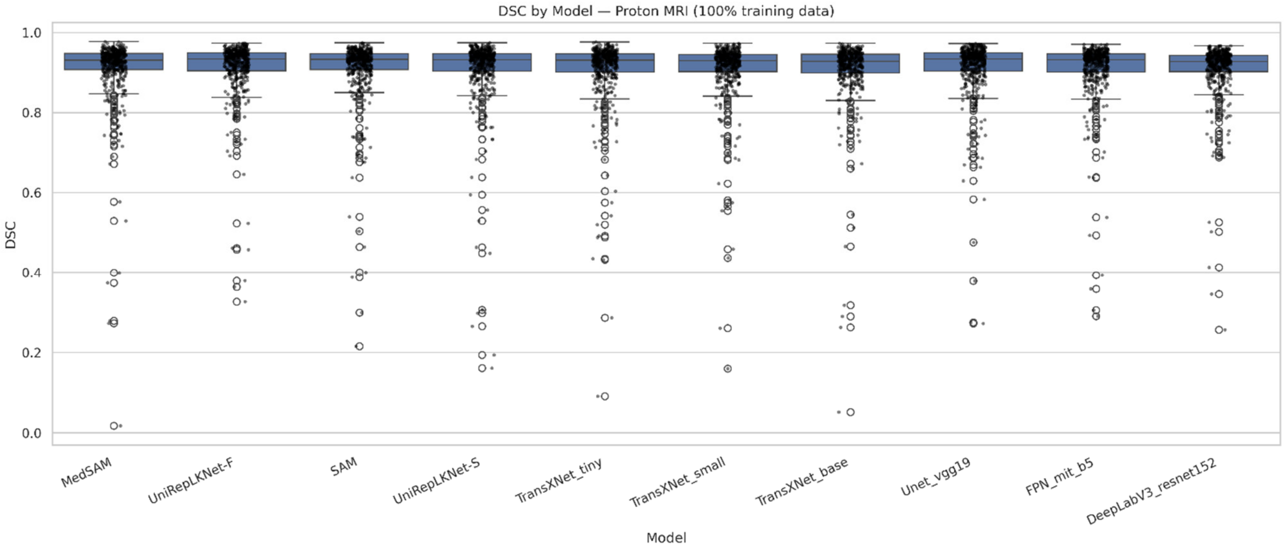

Figure S1. Boxplot showing Dice Similarity Coefficient (DSC) values for proton MRI segmentation using full training data across foundational models (SAM, MedSAM), advanced architectures (UniRepLKNet-F, UniRepLKNet-S, TransXNet variants), and traditional models (UNet-VGG19, FPN-MIT-B5, DeepLabV3-ResNet152). Kruskal-Wallis test revealed minimal differences (H=17.796, p=0.038), with no significant pairwise differences detected (all p>0.01) after Bonferroni correction. All three architectural paradigms demonstrated statistically equivalent performance under optimal data conditions, with DSC values ranging from 0.907-0.913 across all models, indicating that architectural advantages become apparent only under data constraints.

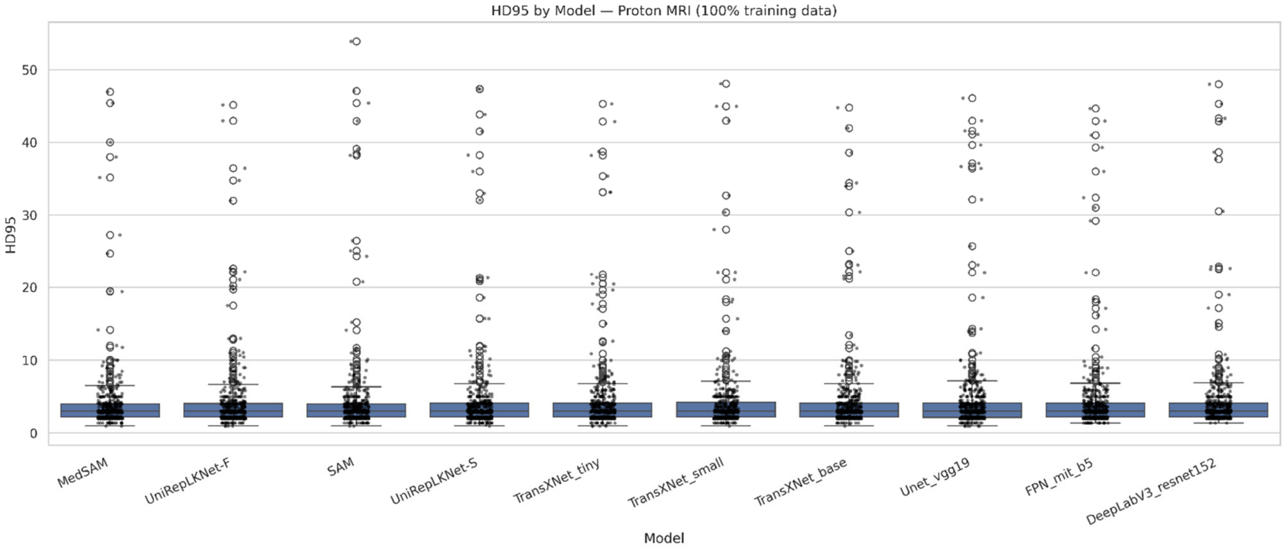

Figure S2. Boxplot showing 95th percentile Hausdorff Distance (HD95) values for proton MRI segmentation using full training data across all model architectures. Statistical analysis revealed no significant differences between any model pairs (Kruskal-Wallis H=8.645, p=0.471),

with HD95 values ranging from 3.996-4.375 across foundational, advanced, and traditional models. This convergent boundary accuracy performance under full data conditions demonstrates that specialized imaging benefits emerge primarily when training data becomes limited.

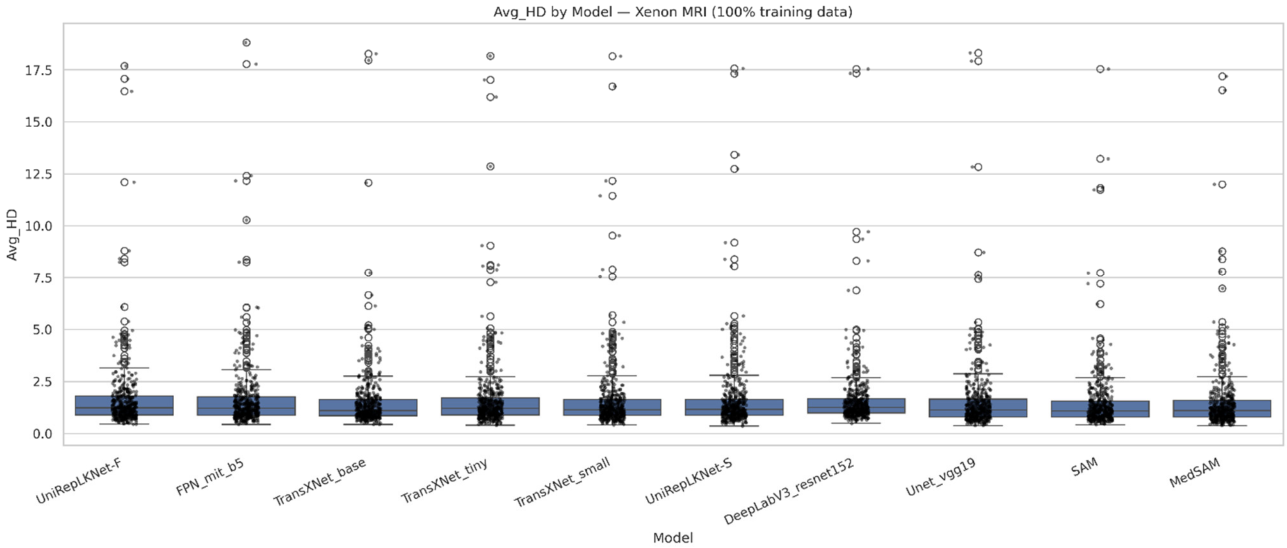

Figure S3. Boxplot showing Average Hausdorff Distance (Avg_HD) values for proton MRI segmentation using full training data. Despite a significant Kruskal-Wallis test (H=17.530, p=0.041), no pairwise comparisons reached significance after Bonferroni correction (all p>0.01). Foundational models (MedSAM: 1.315, SAM: 1.355), advanced models (UniRepLKNet-S: 1.259, TransXNet variants: 1.297-1.346), and traditional models (1.328-1.358) demonstrated equivalent geometric precision under optimal data conditions.

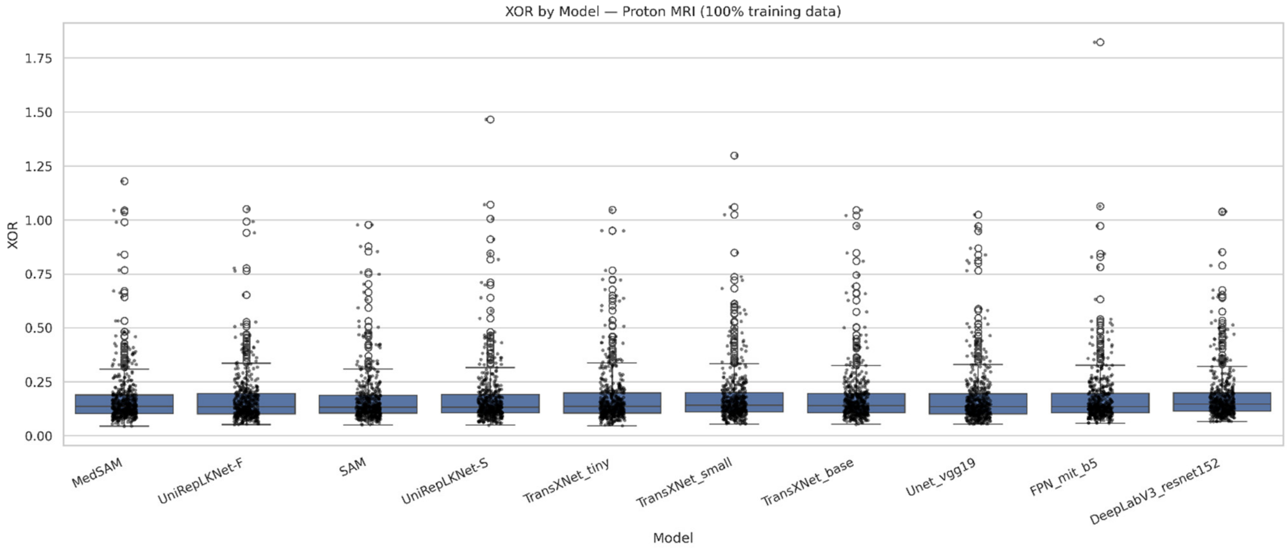

Figure S4. Boxplot showing XOR error metric values for proton MRI segmentation using full training data. While the Kruskal-Wallis test was significant (H=17.994, p=0.035), no pairwise

differences survived Bonferroni correction (all p>0.01). All architectural approaches achieved comparable pixel-wise accuracy (XOR range: 0.169-0.182), establishing baseline equivalence before data reduction scenarios reveal architectural resilience differences.

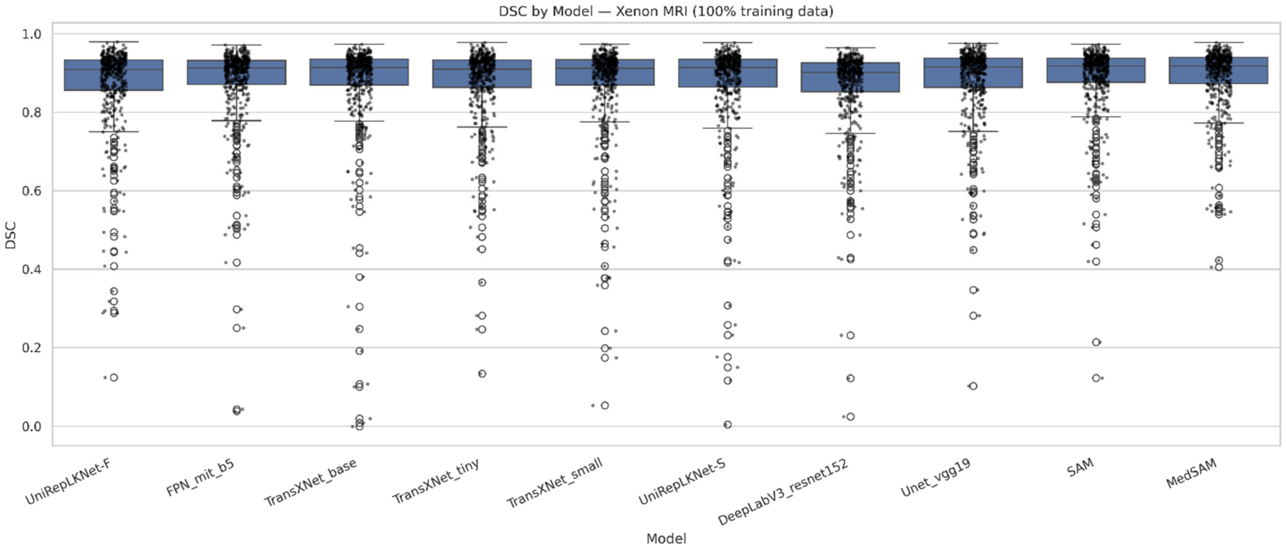

Figure S5. Boxplot showing Dice Similarity Coefficient (DSC) values for hyperpolarized gas MRI segmentation using full training data. Despite significant overall differences (Kruskal-Wallis H=47.948, p<0.001), no pairwise comparisons survived Bonferroni correction (all p>0.01), indicating statistical equivalence across foundational models (MedSAM: 0.891, SAM: 0.887), advanced models (UniRepLKNet variants: 0.872-0.873, TransXNet variants: 0.874-0.877), and traditional models (0.866-0.881).

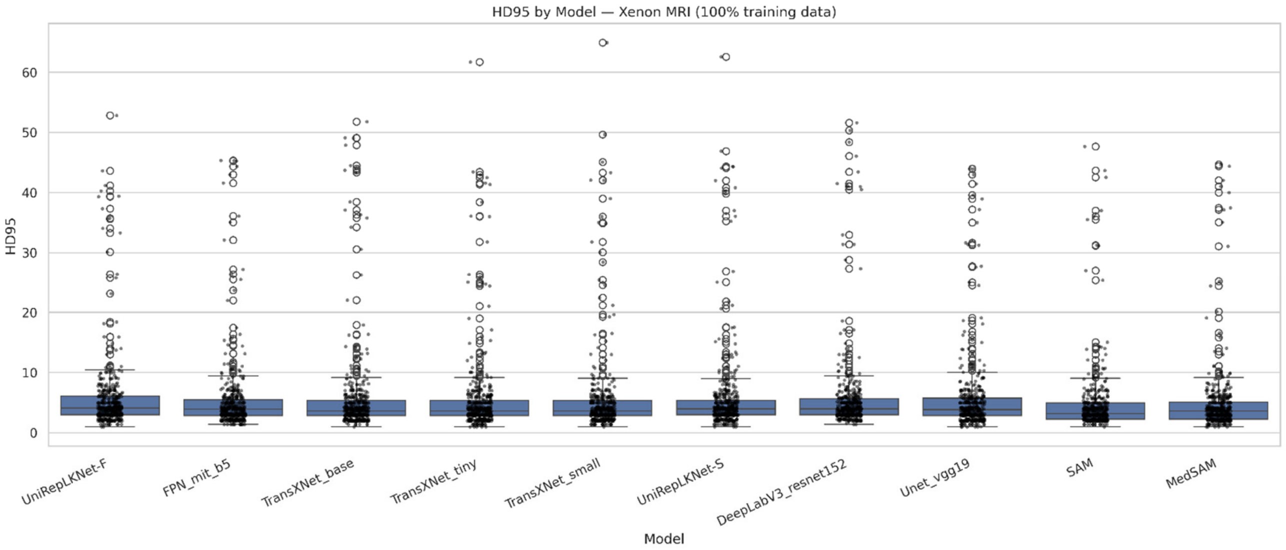

Figure S6. Boxplot showing 95th percentile Hausdorff Distance (HD95) values for hyperpolarized gas MRI segmentation using full training data. Statistical analysis revealed no

significant differences between model pairs after Bonferroni correction (all p>0.01), with HD95 values ranging from 4.667-5.765. The convergent performance across architectural paradigms under full data conditions contrasts with the pronounced divergence observed under data constraints.

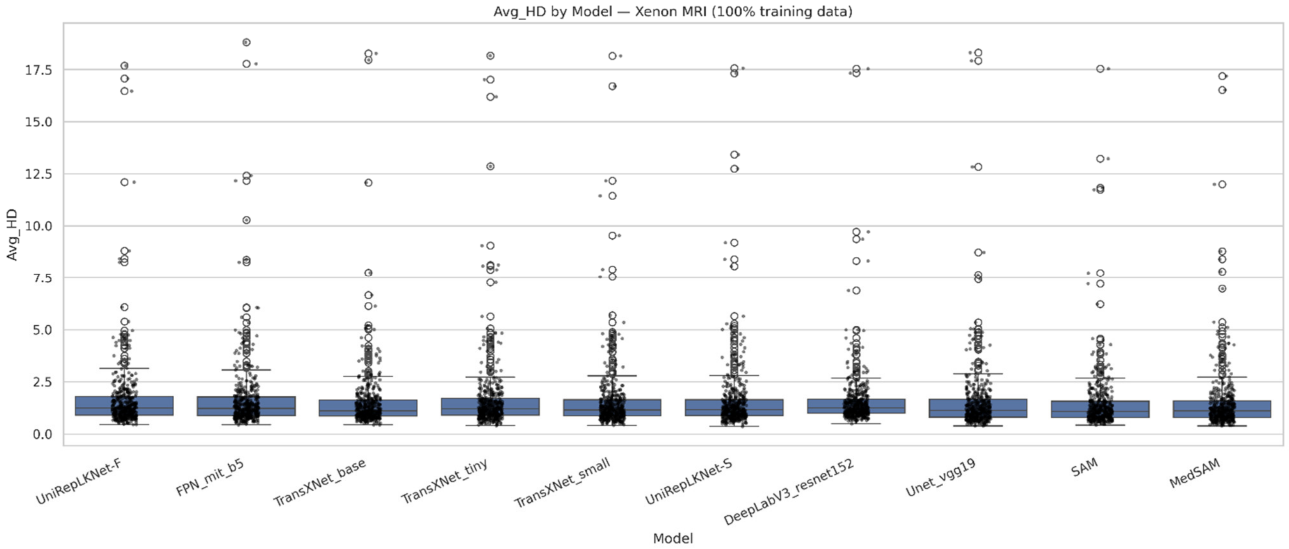

Figure S7. Boxplot showing Average Hausdorff Distance (Avg_HD) values for hyperpolarized gas MRI segmentation using full training data. Despite significant overall differences (Kruskal-Wallis H=48.084, p<0.001), no pairwise comparisons survived Bonferroni correction (all p>0.01). All architectural approaches demonstrated comparable geometric precision (Avg_HD range: 1.435-1.659), establishing equivalent baseline performance.

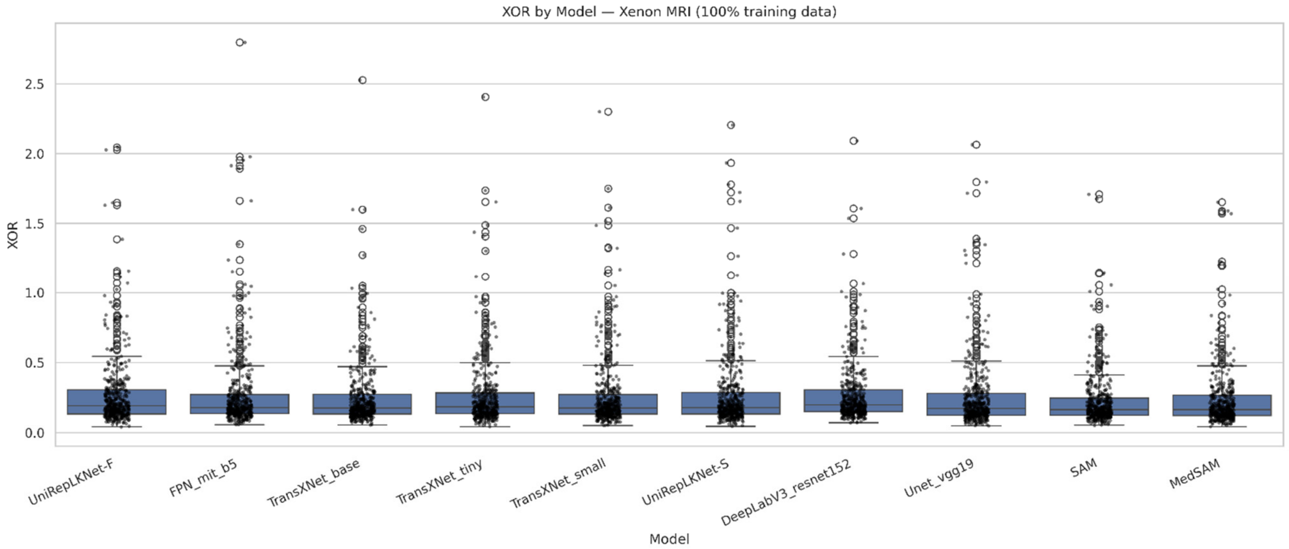

Figure S8. Boxplot showing XOR error metric values for hyperpolarized gas MRI segmentation using full training data. Statistical analysis revealed no significant pairwise differences after Bonferroni correction (all p>0.01), with XOR values ranging from 0.227-0.272 across all architectural paradigms, confirming equivalent pixel-wise accuracy under optimal data conditions.

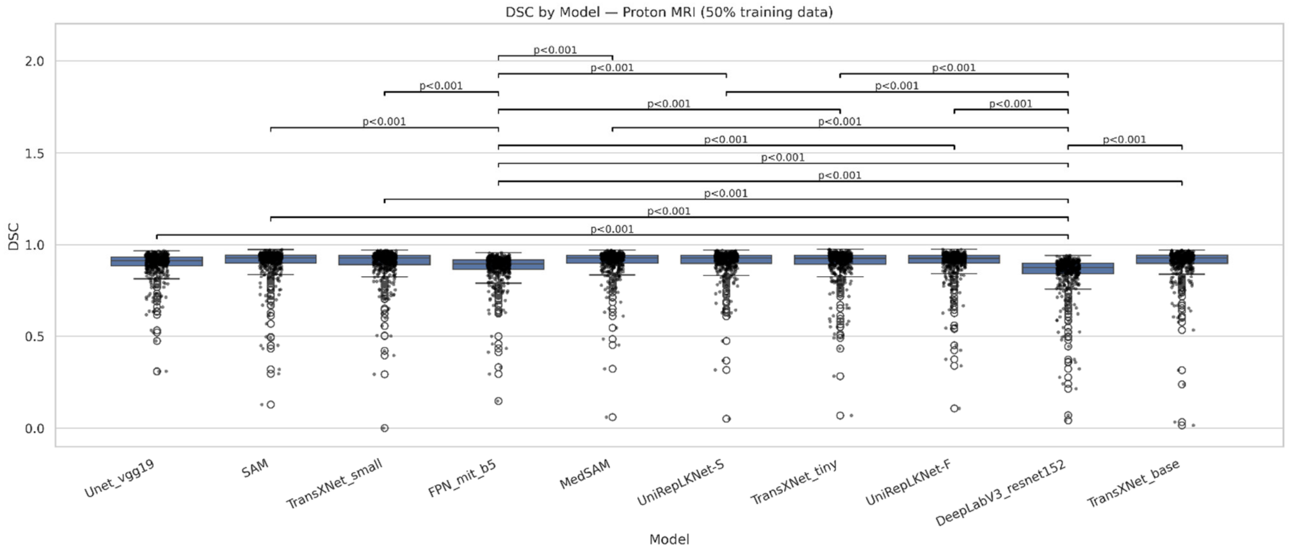

Figure S9. Boxplot showing Dice Similarity Coefficient (DSC) values for proton MRI with moderate data reduction (50% training data). Initial performance divergence emerged (Kruskal-Wallis H=636.108, $p<0.001$), with foundational models (MedSAM: 0.908, SAM: 0.904) and advanced models (UniRepLKNet-F: 0.904, UniRepLKNet-S: 0.907, TransXNet variants: 0.901-0.903) maintaining near-baseline performance. Traditional models showed early degradation: DeepLabV3-ResNet152 (0.846), FPN-MIT-B5 (0.875), UNet-VGG19 (0.896). Statistical analysis revealed significant differences between DeepLabV3-ResNet152 and all other models (all $p<0.001$), while foundational and advanced models demonstrated equivalent robustness (all $p>0.01$).

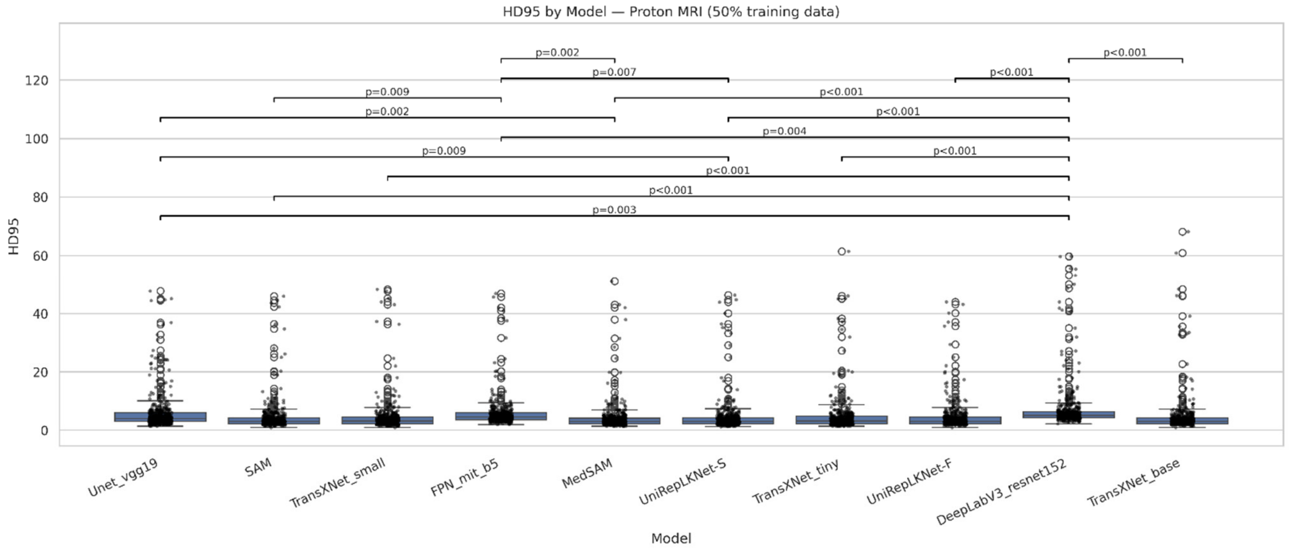

Figure S10. Boxplot showing 95th percentile Hausdorff Distance (HD95) values for proton MRI with moderate data reduction (50% training data). Boundary accuracy analysis (Kruskal-Wallis H=670.675, $p<0.001$) revealed that foundational models (MedSAM: 4.217, SAM: 4.382) and advanced models (UniRepLKNet-S: 4.347, TransXNet variants: 4.498-4.679) maintained precise

boundary delineation. Traditional models showed varying degradation: DeepLabV3-ResNet152 (7.284), FPN-MIT-B5 (5.794), UNet-VGG19 (5.766).

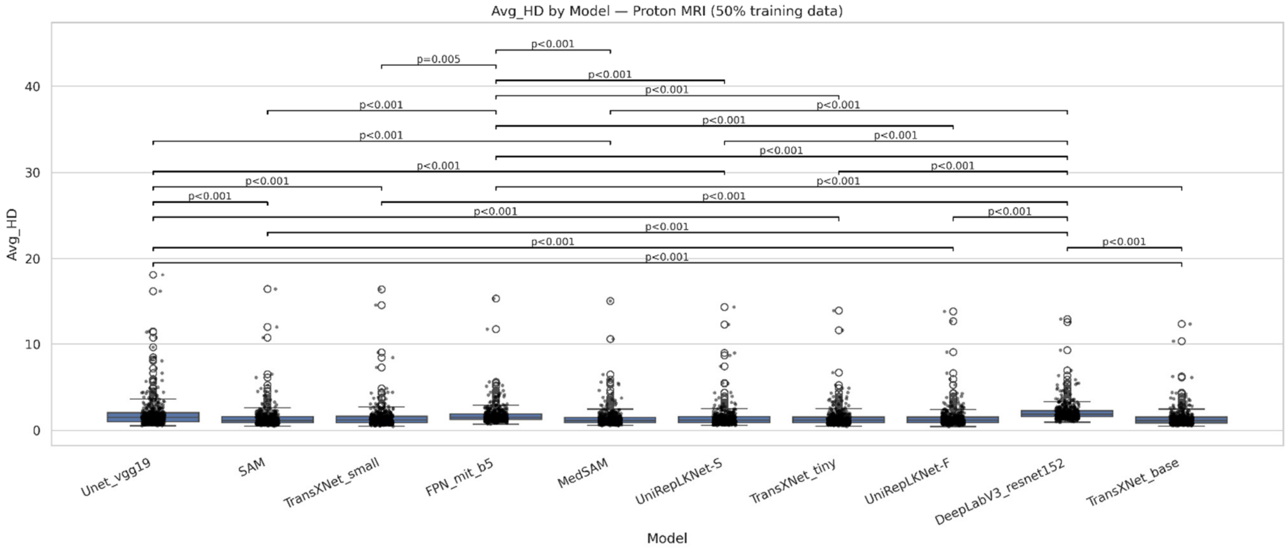

Figure S11. Boxplot showing Average Hausdorff Distance (Avg_HD) values for proton MRI with moderate data reduction (50% training data). Geometric precision analysis (Kruskal-Wallis H=668.608, p<0.001) demonstrated that foundational models (MedSAM: 1.373, SAM: 1.413) and advanced models (UniRepLKNet-S: 1.418, TransXNet variants: 1.343-1.457) maintained excellent geometric accuracy. Traditional models showed increased geometric errors: DeepLabV3-ResNet152 (2.122), UNet-VGG19 (1.894), FPN-MIT-B5 (1.763).

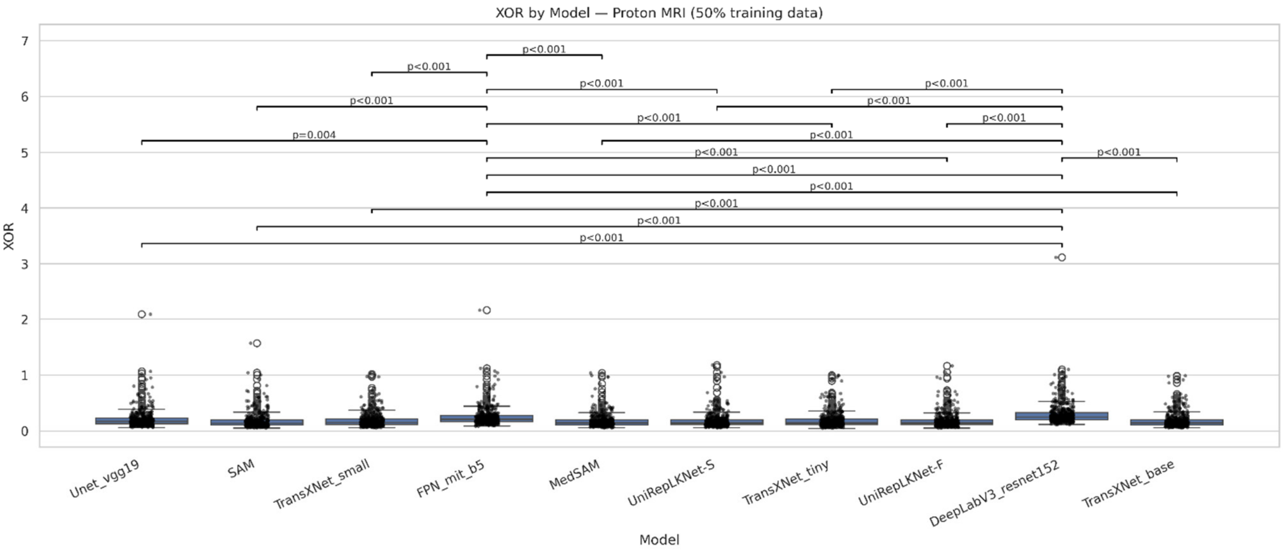

Figure S12. Boxplot showing XOR error metric values for proton MRI with moderate data reduction (50% training data). Pixel-wise accuracy analysis (Kruskal-Wallis H=627.367, p<0.001) revealed that foundational models (MedSAM: 0.180, SAM: 0.184) and advanced models (UniRepLKNet-S: 0.183, TransXNet variants: 0.185-0.190) maintained low error rates. Traditional models exhibited increased pixel-wise errors: DeepLabV3-ResNet152 (0.300), FPN-MIT-B5 (0.251), UNet-VGG19 (0.212).

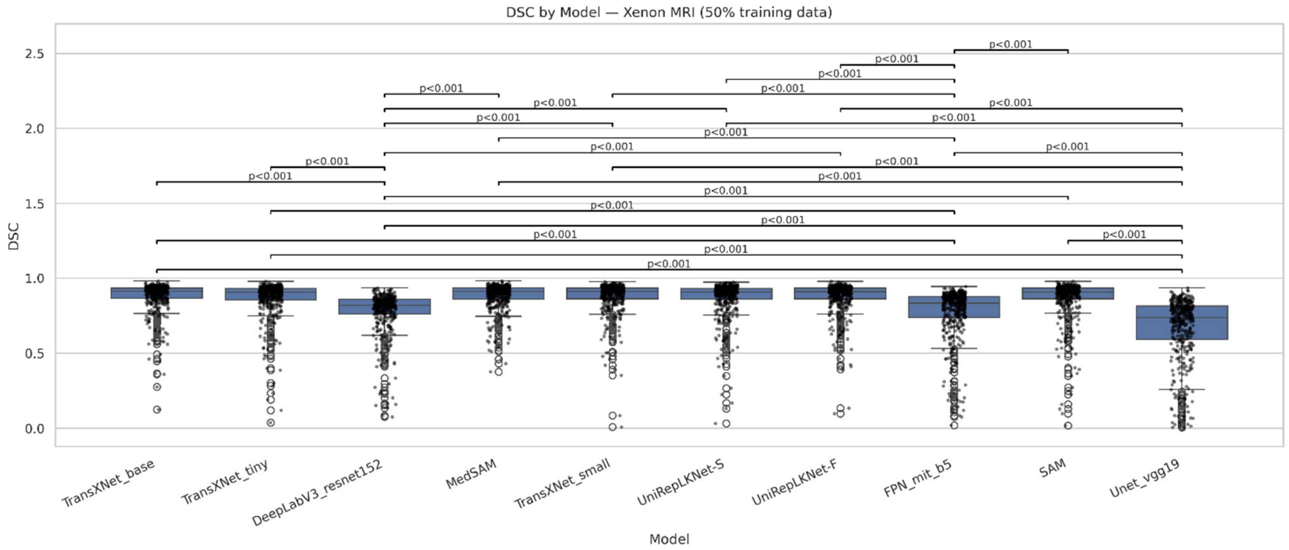

Figure S13. Boxplot showing Dice Similarity Coefficient (DSC) values for hyperpolarized gas MRI with moderate data reduction (50% training data). Pronounced architectural hierarchy emerged earlier in specialized imaging (Kruskal-Wallis H=1227.584, p<0.001), with foundational models (MedSAM: 0.881, SAM: 0.865) and advanced models (UniRepLKNet-F: 0.874, UniRepLKNet-S: 0.867, TransXNet variants: 0.867-0.879) maintaining robust performance. Traditional models experienced substantial degradation: catastrophic failure of UNet-VGG19 (0.666), severe decline in DeepLabV3-ResNet152 (0.775) and FPN-MIT-B5 (0.772).

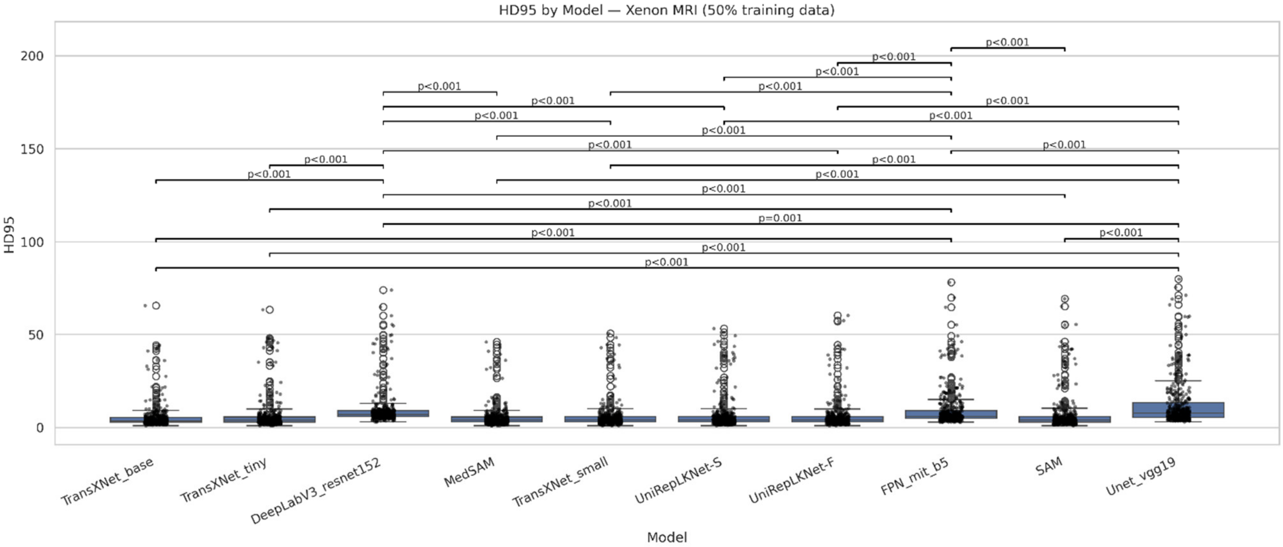

Figure S14. Boxplot showing 95th percentile Hausdorff Distance (HD95) values for hyperpolarized gas MRI with moderate data reduction (50% training data). Boundary accuracy analysis revealed dramatic divergence (Kruskal-Wallis H=982.504, p<0.001), with foundational models (MedSAM: 5.347, SAM: 6.346) and advanced models (UniRepLKNet-S: 6.172, TransXNet variants: 5.305-5.870) maintaining acceptable boundary precision. Traditional models showed severe degradation: UNet-VGG19 (12.167), DeepLabV3-ResNet152 (9.728), FPN-MIT-B5 (9.235).

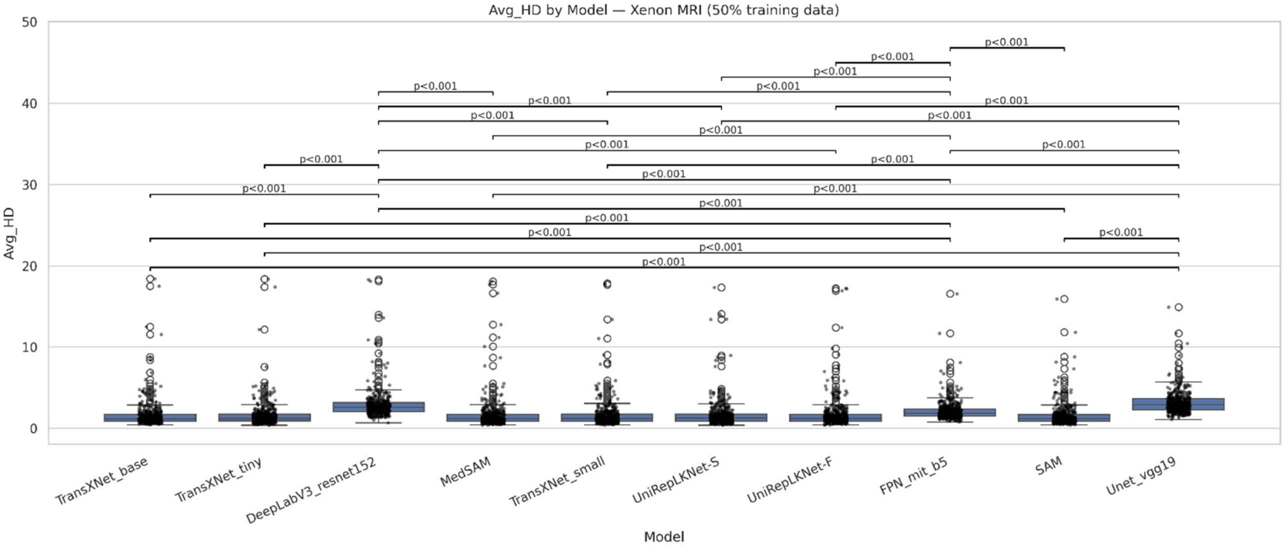

Figure S15. Boxplot showing Average Hausdorff Distance (Avg_HD) values for hyperpolarized gas MRI with moderate data reduction (50% training data). Geometric precision analysis (Kruskal-Wallis H=1315.198, p<0.001) demonstrated that foundational models (MedSAM: 1.612, SAM: 1.544) and advanced models (UniRepLKNet-S: 1.628, TransXNet variants: 1.583-1.649) maintained superior

geometric accuracy. Traditional models exhibited substantial increases in geometric errors: UNet-VGG19 (3.164), DeepLabV3-ResNet152 (2.939), FPN-MIT-B5 (2.167).

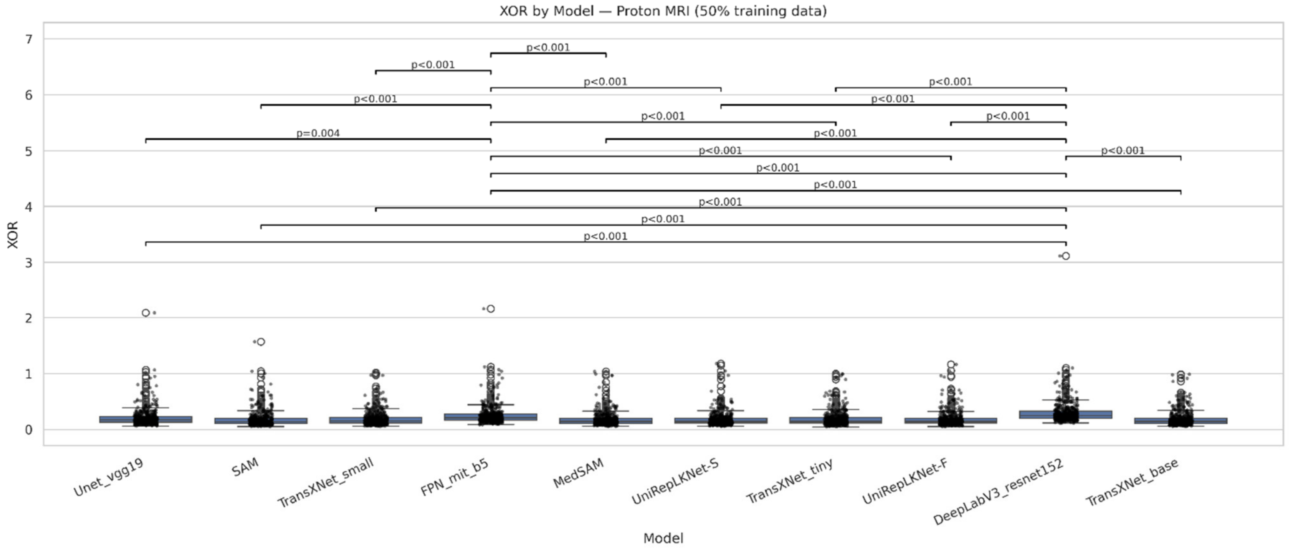

Figure S16. Boxplot showing XOR error metric values for hyperpolarized gas MRI with moderate data reduction (50% training data). Pixel-wise accuracy analysis (Kruskal-Wallis H=1058.358, p<0.001) revealed that foundational models (MedSAM: 0.262, SAM: 0.258) and advanced models (UniRepLKNet-S: 0.267, TransXNet variants: 0.256-0.265) maintained reasonable error rates. Traditional models showed dramatic increases: DeepLabV3-ResNet152 (0.512), UNet-VGG19 (0.488), FPN-MIT-B5 (0.372).

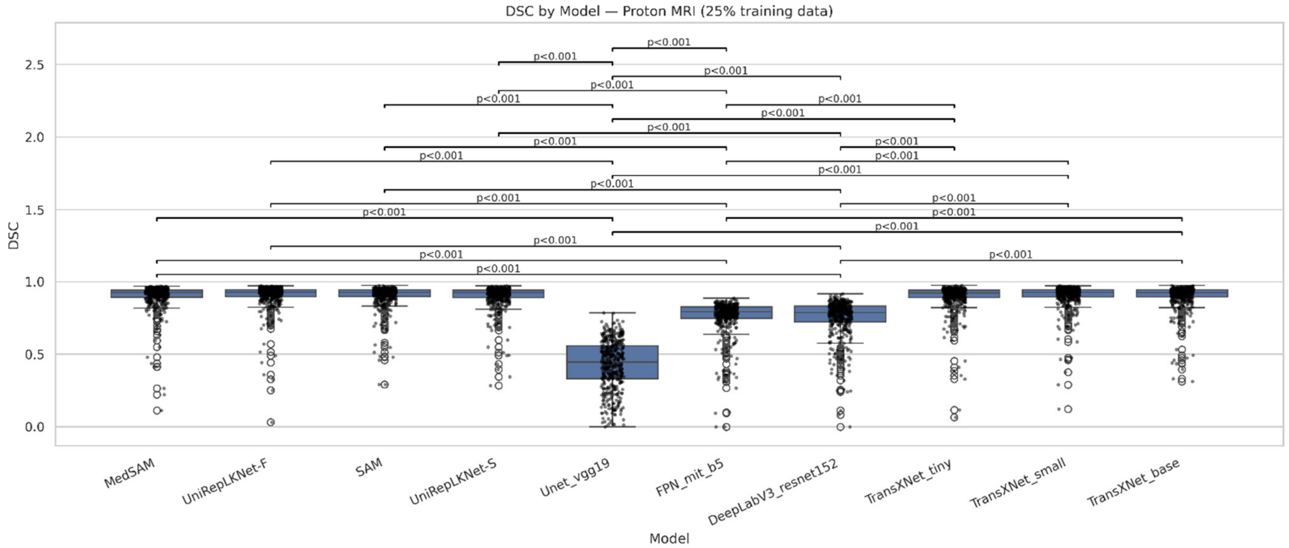

Figure S17. Boxplot showing Dice Similarity Coefficient (DSC) values for proton MRI with significant data reduction (25% training data). Dramatic architectural divergence emerged (Kruskal-Wallis H=2366.272, p<0.001), with foundational models (MedSAM: 0.901, SAM: 0.905) and advanced models (UniRepLKNet-F: 0.905, UniRepLKNet-S: 0.900, TransXNet variants: 0.897-0.904) maintaining near-optimal performance. Traditional models experienced severe degradation: catastrophic failure of UNet-VGG19 (0.428), substantial decline in DeepLabV3-ResNet152 (0.750) and FPN-MIT-B5 (0.758). Statistical analysis confirmed equivalent performance between foundational and advanced models (all p>0.01) while both groups significantly outperformed traditional approaches (all p<0.001).

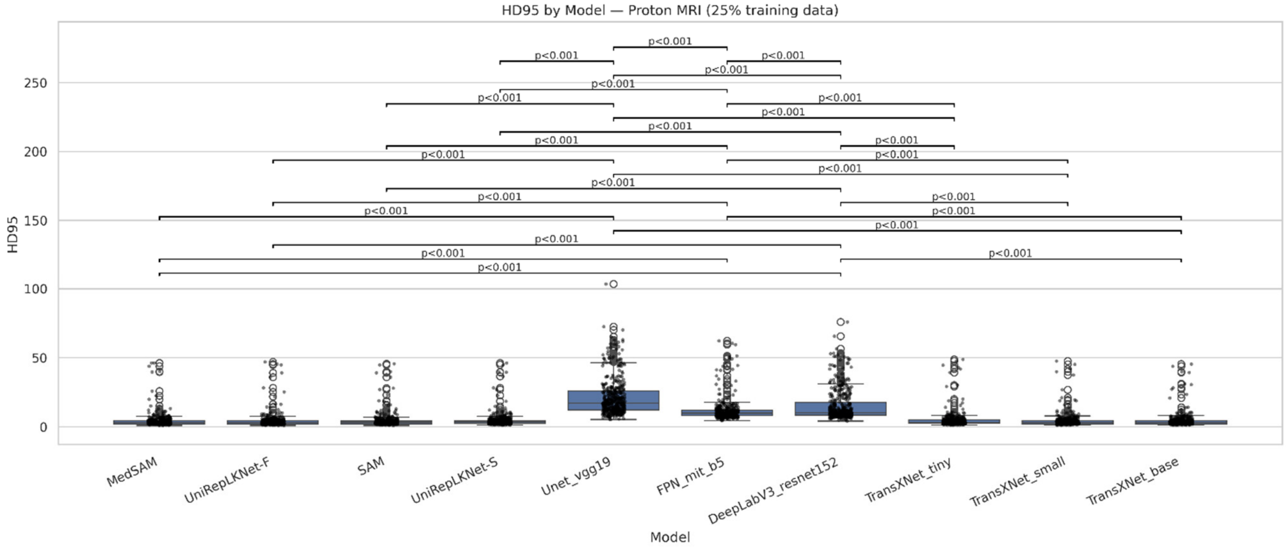

Figure S18. Boxplot showing 95th percentile Hausdorff Distance (HD95) values for proton MRI with significant data reduction (25% training data). Foundational and advanced models maintained superior boundary accuracy (Kruskal-Wallis H=2373.798, p<0.001) with HD95 values of 4.31-4.65, while traditional models showed dramatic degradation: UNet-VGG19 (21.40), DeepLabV3-ResNet152 (15.28), FPN-MIT-B5 (11.78). Both foundational and advanced architectural groups demonstrated statistically equivalent boundary precision (all p>0.01) while significantly outperforming traditional approaches (all p<0.001).

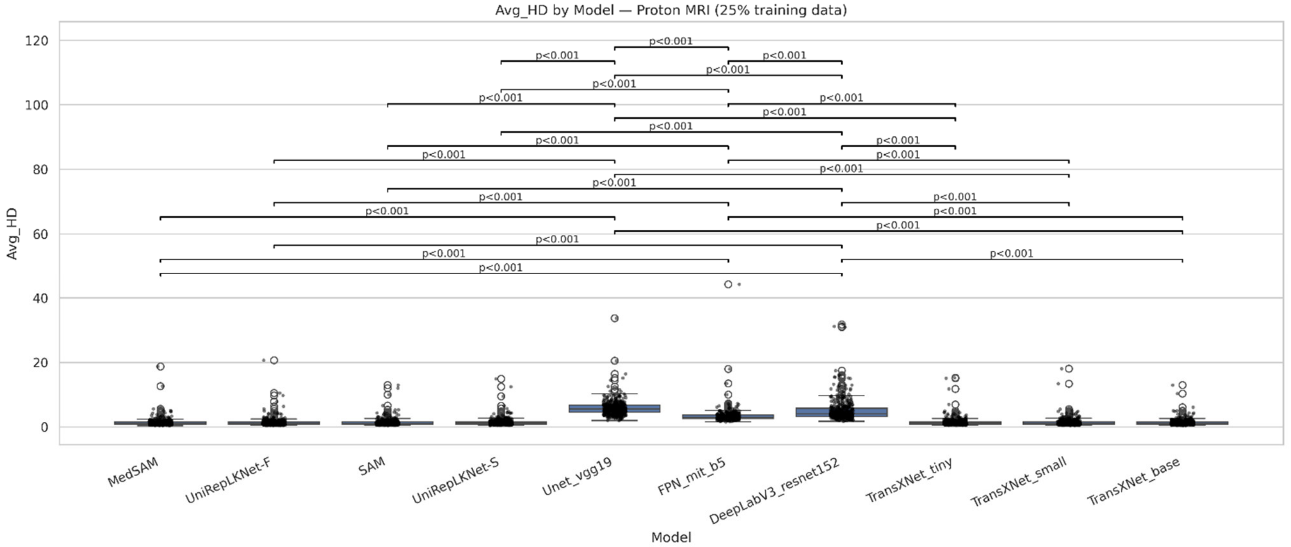

Figure S19. Boxplot showing Average Hausdorff Distance (Avg_HD) values for proton MRI with significant data reduction (25% training data). The superior geometric precision (Kruskal-Wallis H=2492.355, p<0.001) of foundational models (MedSAM: 1.409, SAM: 1.403) and advanced models (UniRepLKNet variants: 1.417-1.474, TransXNet variants: 1.399-1.484) contrasts sharply with traditional model degradation: DeepLabV3-ResNet152 (5.107), UNet-VGG19 (5.887), FPN-MIT-B5 (3.431). Statistical equivalence between foundational and advanced approaches (all p>0.01) coupled with their significant superiority over traditional models (all p<0.001) demonstrates the critical importance of large effective receptive fields.

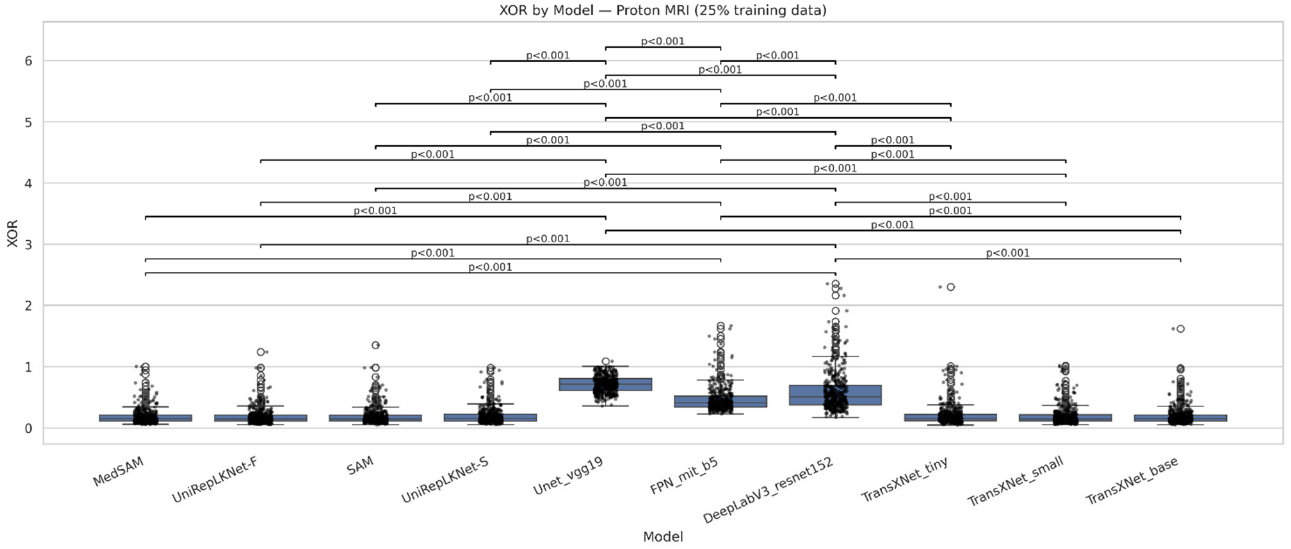

Figure S20. Boxplot showing XOR error metric values for proton MRI with significant data reduction (25% training data). Foundational models (MedSAM: 0.186, SAM: 0.185) and advanced models

(UniRepLKNet-F: 0.184, UniRepLKNet-S: 0.192, TransXNet variants: 0.191-0.201) maintained low pixel-wise error rates (Kruskal-Wallis H=2350.866, p<0.001). Traditional models exhibited substantial increases: UNet-VGG19 (0.716), DeepLabV3-ResNet152 (0.591), FPN-MIT-B5 (0.467). The statistical equivalence between foundational and advanced models with their shared superiority over traditional approaches confirms the robustness of architectures incorporating large effective receptive fields.

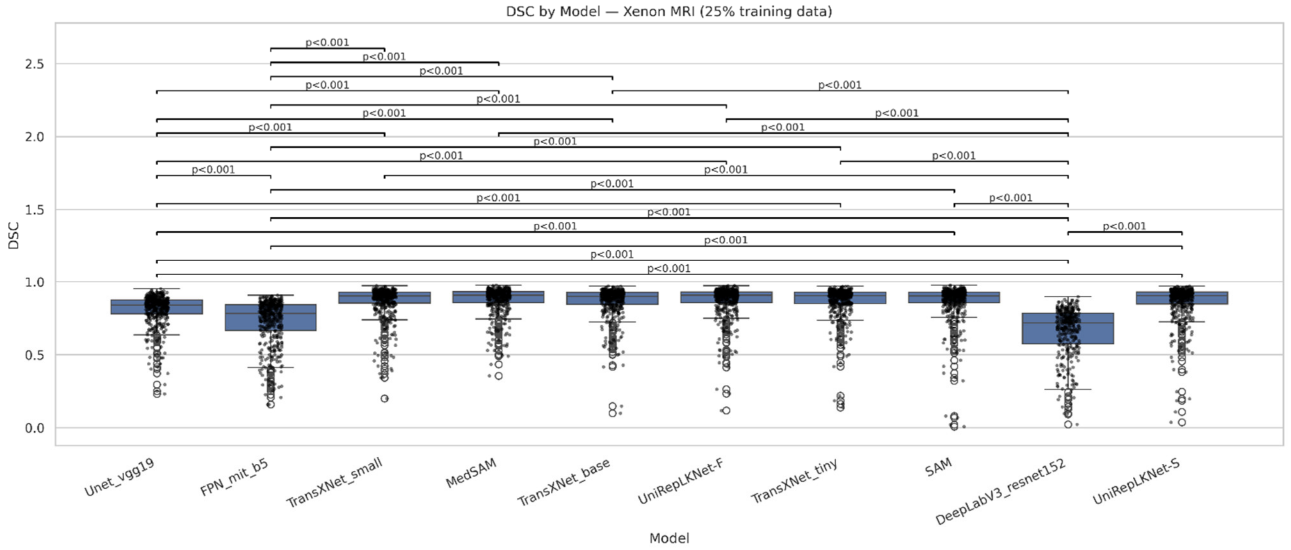

Figure S21. Boxplot showing Dice Similarity Coefficient (DSC) values for hyperpolarized gas MRI with significant data reduction (25% training data). Dramatic performance hierarchy emerged (Kruskal-Wallis H=1278.075, p<0.001), with foundational models (MedSAM: 0.877, SAM: 0.866) and advanced models (UniRepLKNet-F: 0.871, UniRepLKNet-S: 0.865, TransXNet variants: 0.867-0.870) maintaining robust performance above 0.86 DSC. Traditional models showed substantial degradation: UNet-VGG19 (0.811), FPN-MIT-B5 (0.729), DeepLabV3-ResNet152 (0.661). Statistical analysis confirmed equivalent resilience between foundational and advanced architectural paradigms (all p>0.01) while both significantly outperformed traditional approaches (all p<0.001).

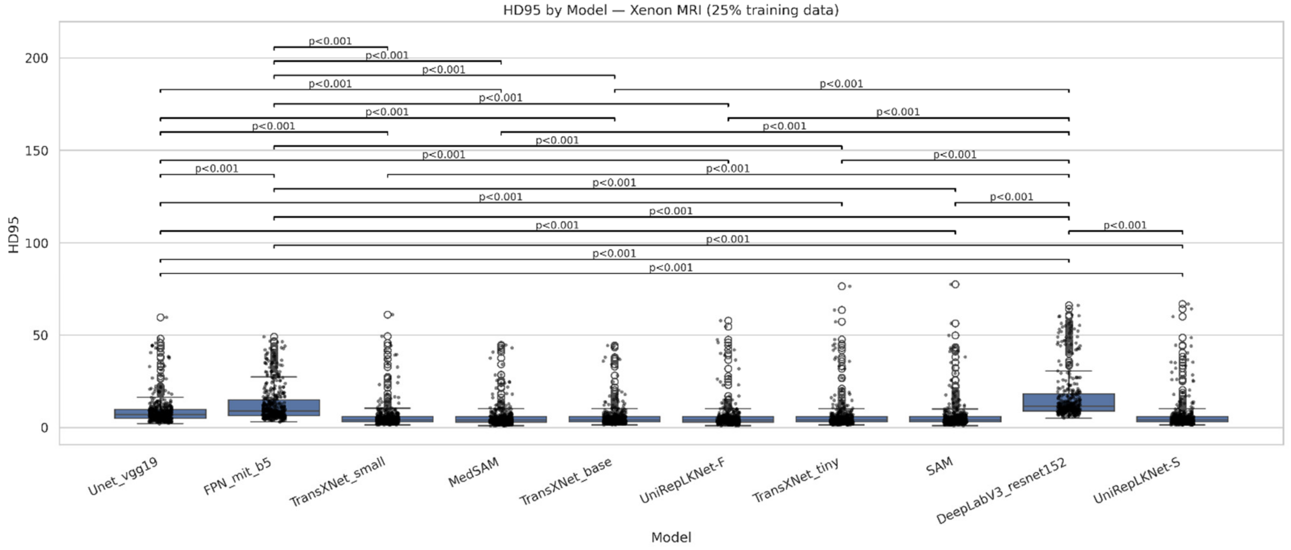

Figure S22. Boxplot showing 95th percentile Hausdorff Distance (HD95) values for hyperpolarized gas MRI with significant data reduction (25% training data). Boundary accuracy analysis (Kruskal-Wallis H=1345.493, p<0.001) revealed that foundational models (MedSAM: 5.511, SAM: 5.883) and advanced models (UniRepLKNet-S: 6.309, TransXNet variants: 5.734-5.971) maintained acceptable boundary precision. Traditional models exhibited severe degradation: DeepLabV3-ResNet152 (17.202), FPN-MIT-B5 (12.768), UNet-VGG19 (8.958), demonstrating 2-3x performance deterioration in boundary accuracy.

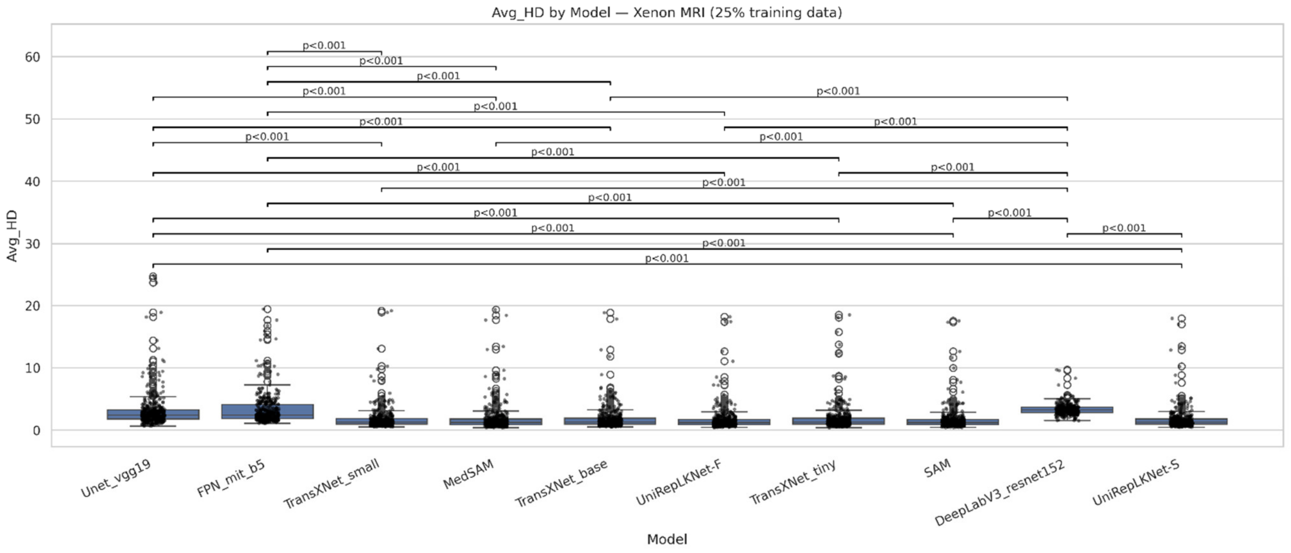

Figure S23. Boxplot showing Average Hausdorff Distance (Avg_HD) values for hyperpolarized gas MRI with significant data reduction (25% training data). Geometric precision analysis (Kruskal-Wallis H=1333.658, p<0.001) demonstrated that foundational models (MedSAM: 1.781, SAM:

1.623) and advanced models (UniRepLKNet-S: 1.692, TransXNet variants: 1.748-1.801) maintained superior geometric accuracy. Traditional models showed substantial degradation: UNet-VGG19 (3.075), FPN-MIT-B5 (3.268), DeepLabV3-ResNet152 (3.365), highlighting the robust geometric precision maintained by advanced architectural paradigms.

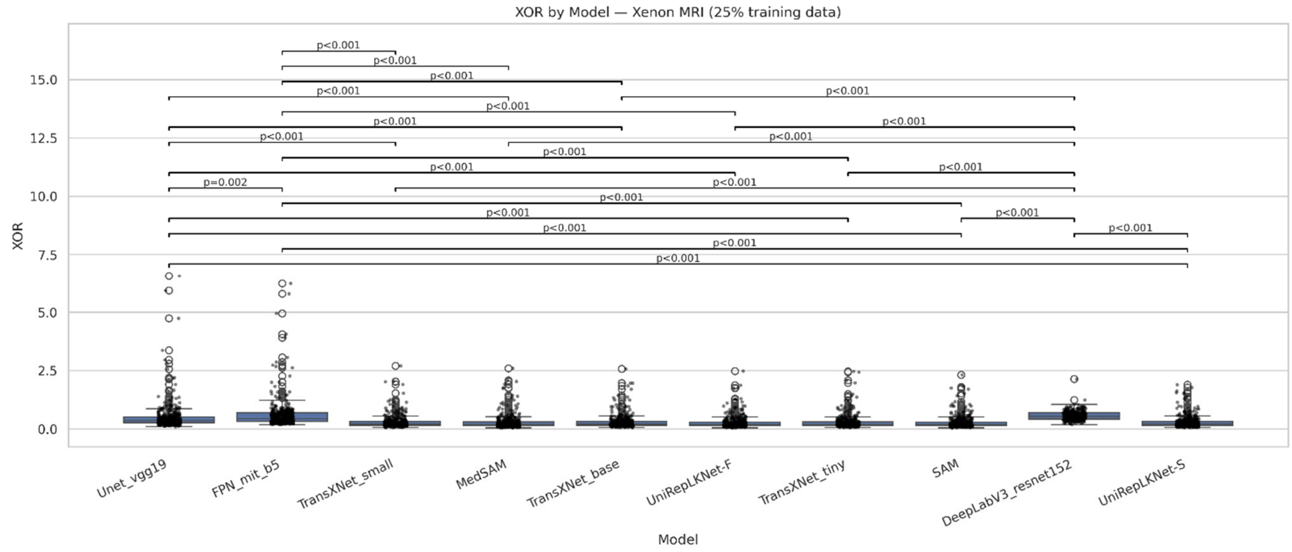

Figure S24. Boxplot showing XOR error metric values for hyperpolarized gas MRI with significant data reduction (25% training data). Pixel-wise accuracy analysis (Kruskal-Wallis H=1196.363, p<0.001) revealed that foundational models (MedSAM: 0.280, SAM: 0.272) and advanced models (UniRepLKNet-F: 0.271, UniRepLKNet-S: 0.282, TransXNet variants: 0.280-0.287) maintained reasonable error rates. Traditional models showed substantial increases: UNet-VGG19 (0.499), FPN-MIT-B5 (0.599), DeepLabV3-ResNet152 (0.565), underscoring the superior pixel-level accuracy of advanced architectural approaches under data constraints.

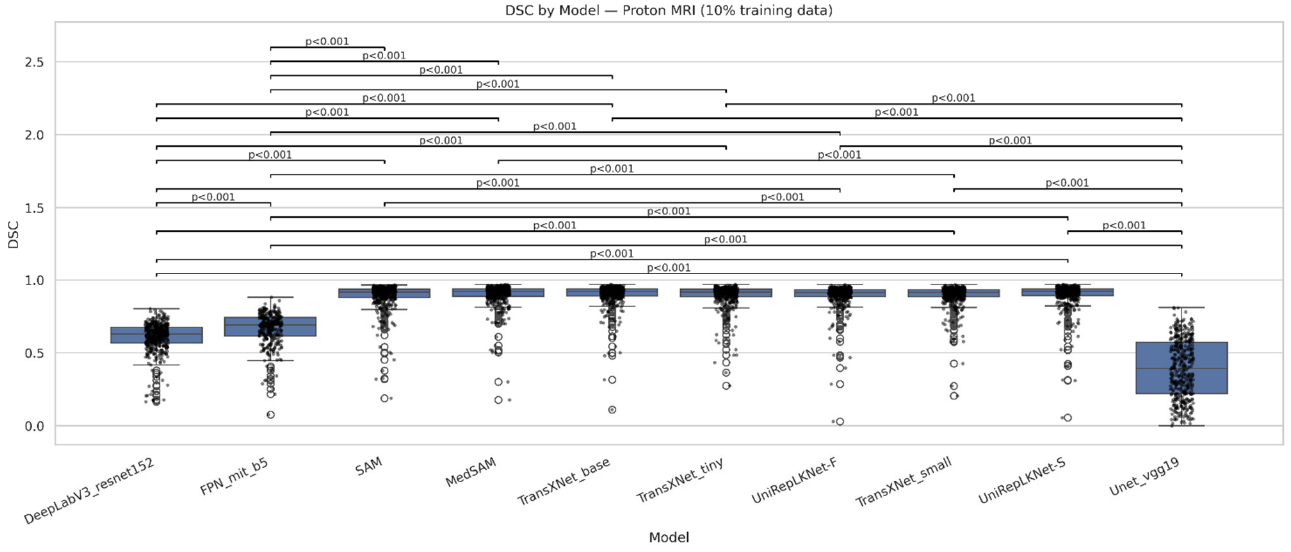

Figure S25. Boxplot showing Dice Similarity Coefficient (DSC) values for proton MRI with extreme data limitation (10% training data). Under these challenging conditions (Kruskal-Wallis H=2472.807, p<0.001), foundational models (MedSAM: 0.901, SAM: 0.892) and advanced models (UniRepLKNet-F: 0.895, UniRepLKNet-S: 0.899, TransXNet variants: 0.896-0.900) demonstrated remarkable stability, maintaining DSC >0.89. Traditional models experienced complete performance collapse: UNet-VGG19 (0.392), DeepLabV3-ResNet152 (0.610), FPN-MIT-B5 (0.665). This extreme scenario definitively establishes the superior data efficiency of architectures incorporating large effective receptive fields.

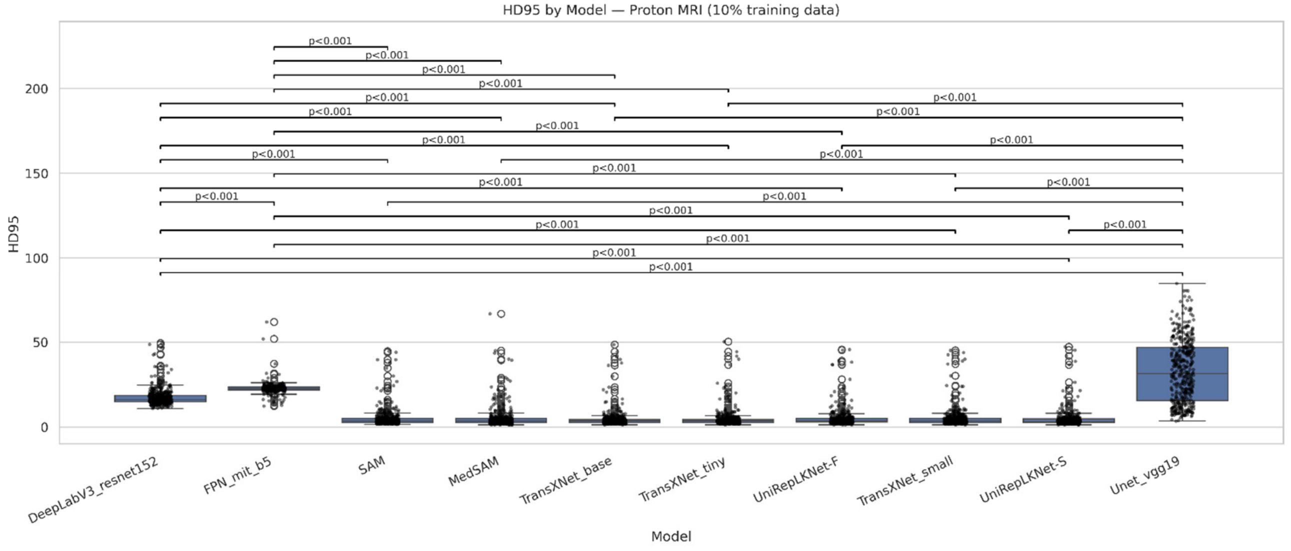

Figure S26. Boxplot showing 95th percentile Hausdorff Distance (HD95) values for proton MRI with extreme data limitation (10% training data). Boundary accuracy analysis (Kruskal-Wallis

H=2411.021, p<0.001) revealed that foundational models (MedSAM: 5.064, SAM: 5.102) and advanced models (UniRepLKNet-S: 4.521, TransXNet variants: 4.583-4.978) maintained excellent boundary precision. Traditional models showed catastrophic degradation: UNet-VGG19 (33.138), FPN-MIT-B5 (22.986), DeepLabV3-ResNet152 (17.491), representing 4-7x performance deterioration in boundary accuracy.

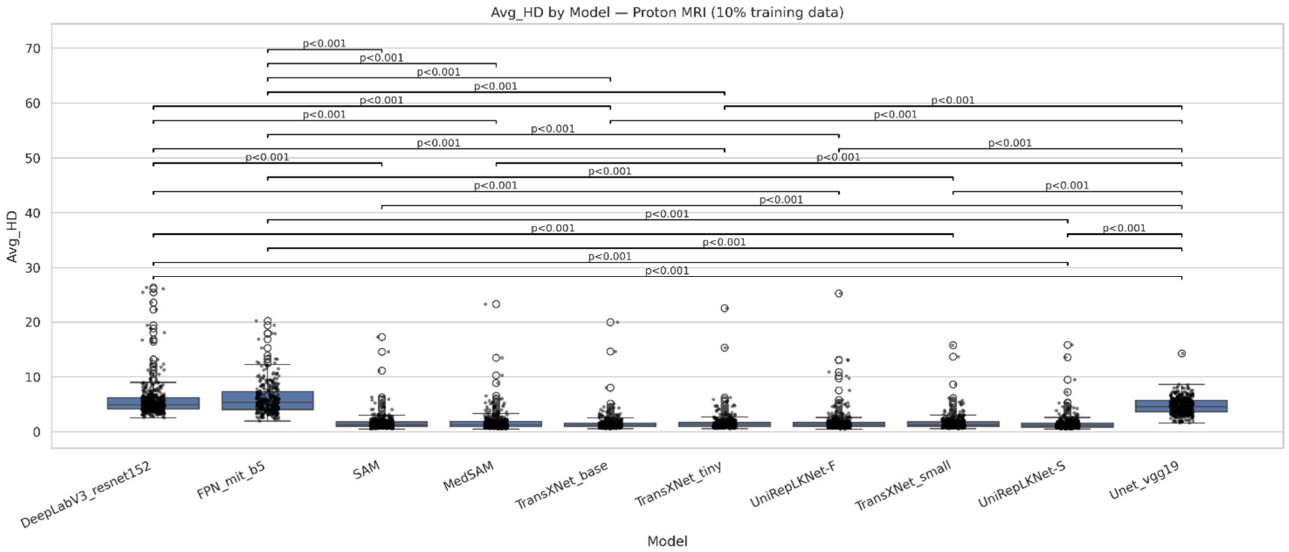

Figure S27. Boxplot showing Average Hausdorff Distance (Avg_HD) values for proton MRI with extreme data limitation (10% training data). Geometric precision analysis (Kruskal-Wallis H=2376.067, p<0.001) demonstrated that foundational models (MedSAM: 1.670, SAM: 1.616) and advanced models (UniRepLKNet-S: 1.477, TransXNet variants: 1.475-1.610) maintained superior geometric accuracy. Traditional models exhibited severe degradation: DeepLabV3-ResNet152 (5.684), FPN-MIT-B5 (6.072), UNet-VGG19 (4.711), confirming the exceptional resilience of advanced architectural approaches under extreme data constraints.

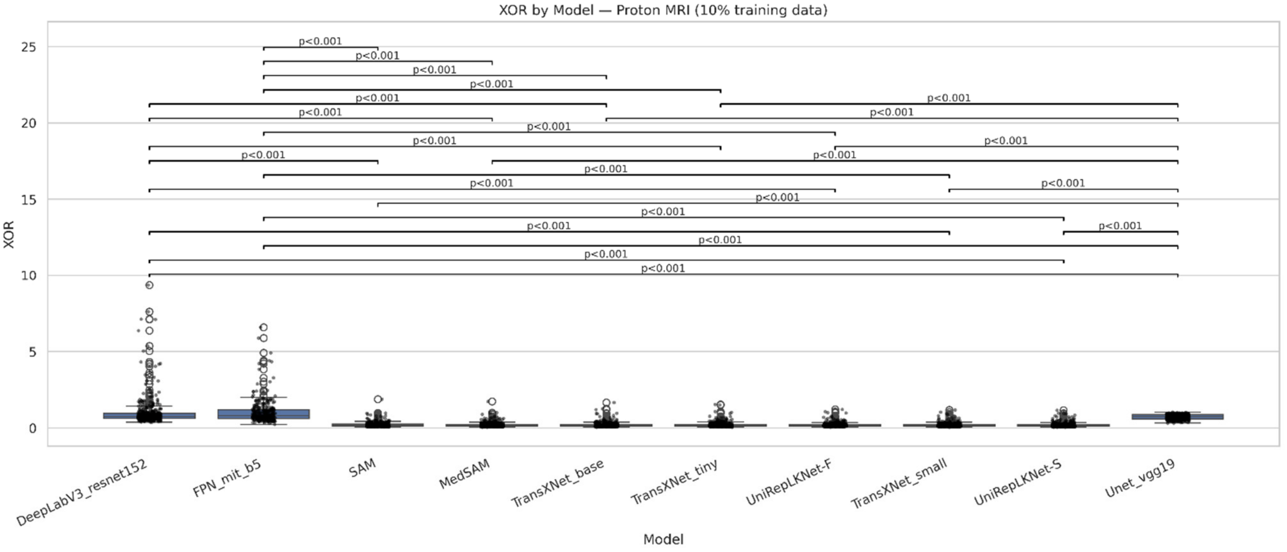

Figure S28. Boxplot showing XOR error metric values for proton MRI with extreme data limitation (10% training data). Pixel-wise accuracy analysis (Kruskal-Wallis H=2449.217, $p<0.001$) revealed that foundational models (MedSAM: 0.202, SAM: 0.211) and advanced models (UniRepLKNet-S: 0.196, TransXNet variants: 0.201-0.208) maintained remarkably low error rates. Traditional models showed dramatic increases: DeepLabV3-ResNet152 (0.989), FPN-MIT-B5 (1.034), UNet-VGG19 (0.740), representing 3-5x deterioration in pixel-wise accuracy.

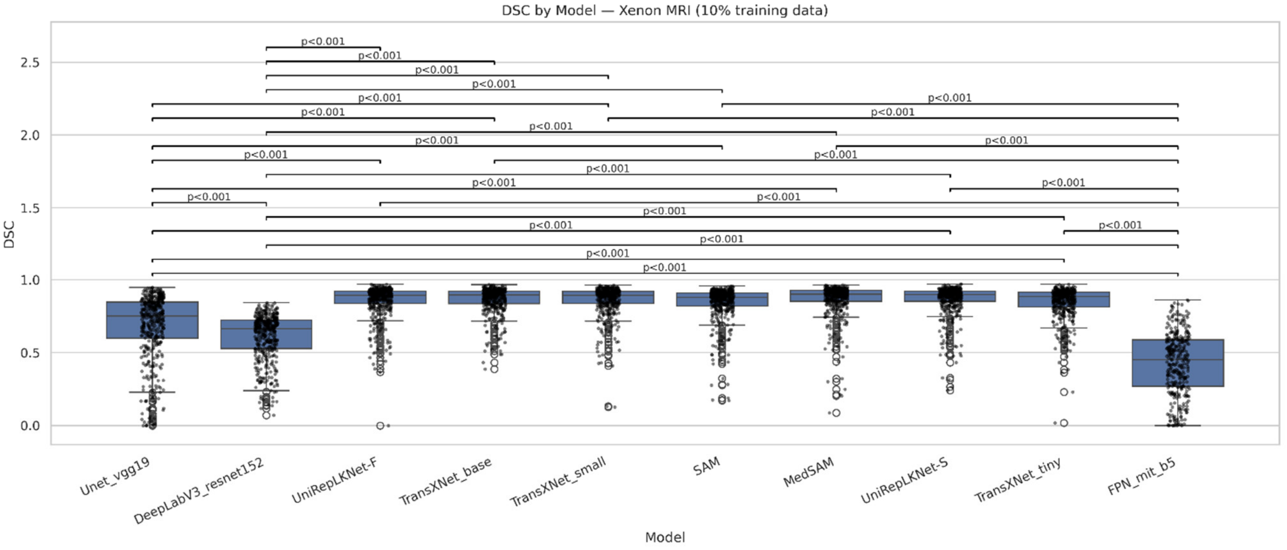

Figure S29. Boxplot showing Dice Similarity Coefficient (DSC) values for hyperpolarized gas MRI with extreme data limitation (10% training data). Even under severe data constraints (Kruskal-Wallis H=1819.049, $p<0.001$), foundational models (MedSAM: 0.867, SAM: 0.841) and advanced models

(UniRepLKNet-F: 0.859, UniRepLKNet-S: 0.865, TransXNet variants: 0.847-0.864) maintained clinically viable performance above 0.84 DSC. Traditional models showed severe degradation: UNet-VGG19 (0.679), DeepLabV3-ResNet152 (0.610), FPN-MIT-B5 (0.423). The sustained equivalence between foundational and advanced approaches validates multiple architectural pathways for robust performance in data-limited scenarios.

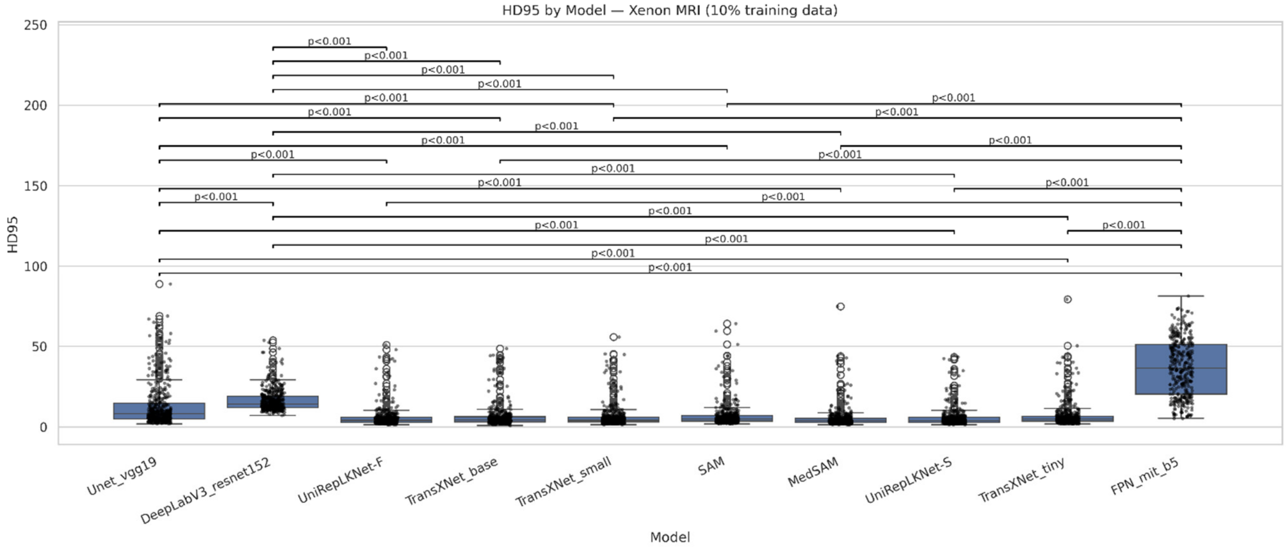

Figure S30. Boxplot showing 95th percentile Hausdorff Distance (HD95) values for hyperpolarized gas MRI with extreme data limitation (10% training data). Boundary accuracy analysis (Kruskal-Wallis H=1788.120, $p<0.001$) revealed that foundational models (MedSAM: 5.504, SAM: 7.044) and advanced models (UniRepLKNet-S: 5.606, TransXNet variants: 6.263-6.904) maintained acceptable boundary precision. Traditional models exhibited catastrophic degradation: FPN-MIT-B5 (36.940), DeepLabV3-ResNet152 (16.481), UNet-VGG19 (12.858), demonstrating the critical importance of advanced architectural designs for boundary delineation under extreme data limitations.

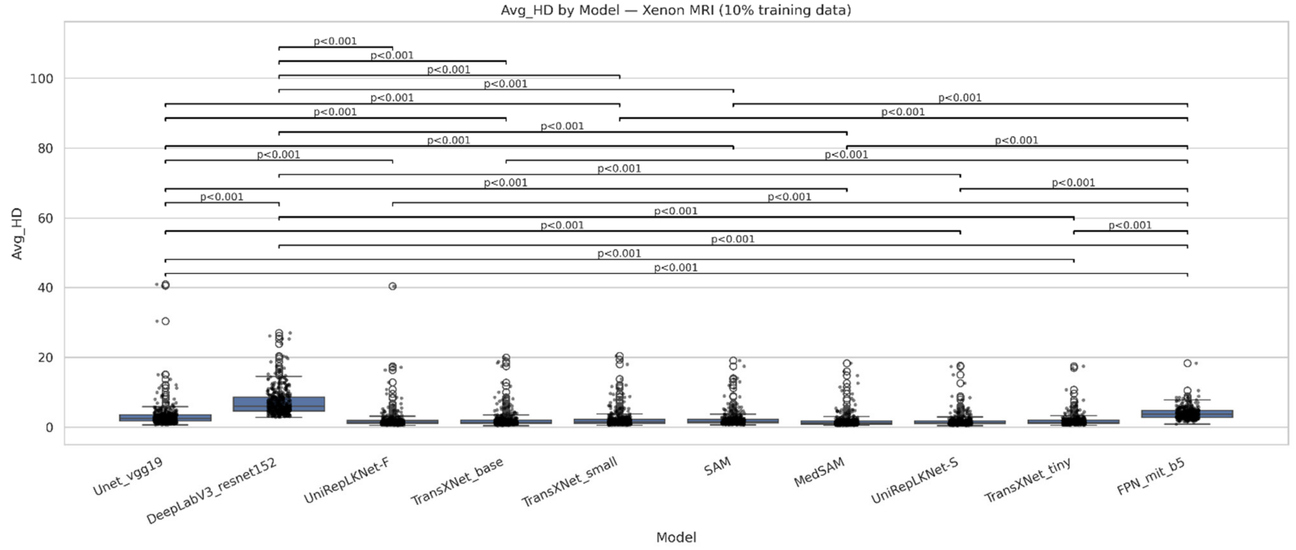

Figure S31. Boxplot showing Average Hausdorff Distance (Avg_HD) values for hyperpolarized gas MRI with extreme data limitation (10% training data). Geometric precision analysis (Kruskal-Wallis H=1764.856, p<0.001) demonstrated that foundational models (MedSAM: 1.719, SAM: 2.105) and advanced models (UniRepLKNet-S: 1.732, TransXNet variants: 1.803-2.108) maintained superior geometric accuracy. Traditional models showed substantial degradation: DeepLabV3-ResNet152 (7.168), FPN-MIT-B5 (4.011), UNet-VGG19 (3.153), confirming the exceptional geometric precision of advanced approaches even under the most challenging data constraints.

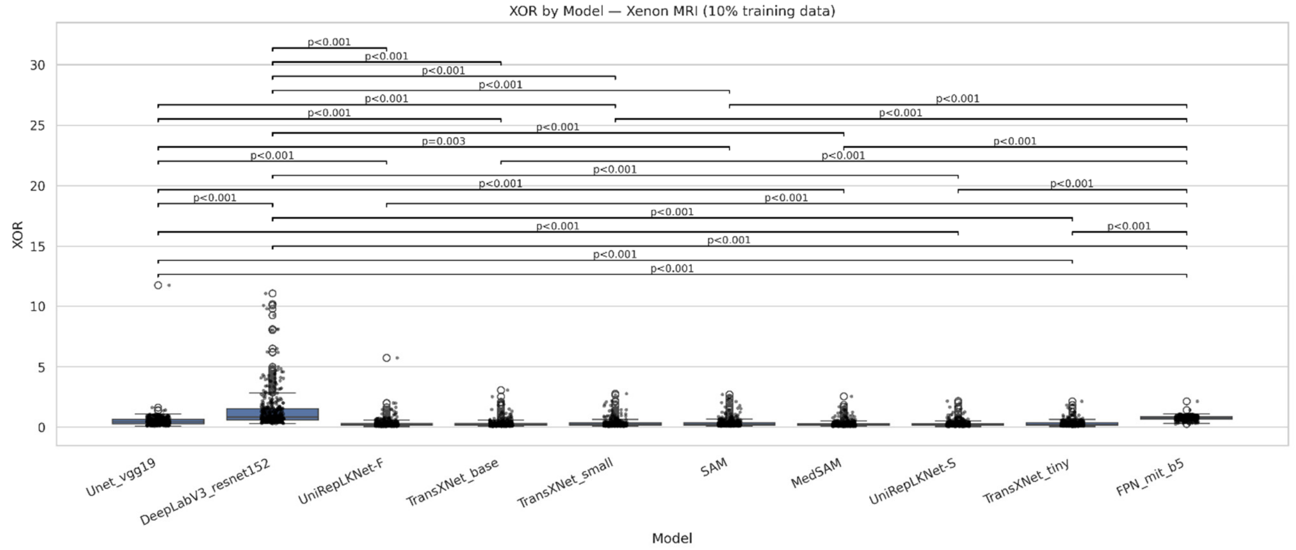

Figure S32. Boxplot showing XOR error metric values for hyperpolarized gas MRI with extreme data limitation (10% training data). Pixel-wise accuracy analysis (Kruskal-Wallis H=1700.129, p<0.001) revealed that foundational models (MedSAM: 0.278, SAM: 0.353) and advanced models

(UniRepLKNet-S: 0.282, TransXNet variants: 0.305-0.320) maintained reasonable error rates. Traditional models exhibited dramatic increases: DeepLabV3-ResNet152 (1.406), FPN-MIT-B5 (0.774), UNet-VGG19 (0.509), representing 2-5x deterioration in pixel-wise accuracy and confirming the superior data efficiency of advanced architectural paradigms.

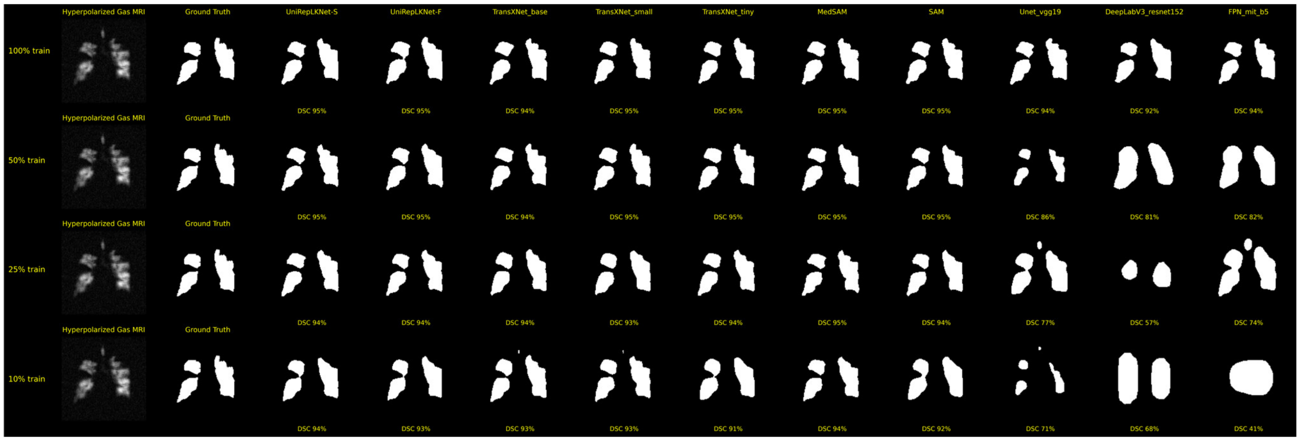

Figure S33. Qualitative comparison of hyperpolarized gas MRI lung segmentation results across progressive data reduction scenarios and model architectures. Representative hyperpolarized gas MRI slice (leftmost column) with corresponding ground truth segmentation (second column) and model predictions across foundational models (MedSAM, SAM), advanced architectures (UniRepLKNet-F, UniRepLKNet-S, TransXNet variants), and traditional models (UNet-VGG19, DeepLabV3-ResNet152, FPN-MIT-B5). White regions represent segmented lung areas with Dice Similarity Coefficient (DSC) values displayed above each result. Progressive data reduction from 100% to 10% training data (top to bottom rows) reveals the superior resilience of foundational and advanced models, which maintain anatomically accurate lung boundaries and high DSC scores (>0.85) even under extreme data constraints. Traditional architectures exhibit progressive degradation in segmentation quality, with irregular boundaries, anatomical inaccuracies, and substantially reduced DSC scores, particularly evident in the 10% training data scenario where UNet-VGG19 and DeepLabV3-ResNet152 produce fragmented or oversimplified segmentations. This visual assessment confirms the quantitative findings demonstrating the critical advantage of architectures incorporating large effective receptive fields for robust performance in data-limited specialized medical imaging applications.

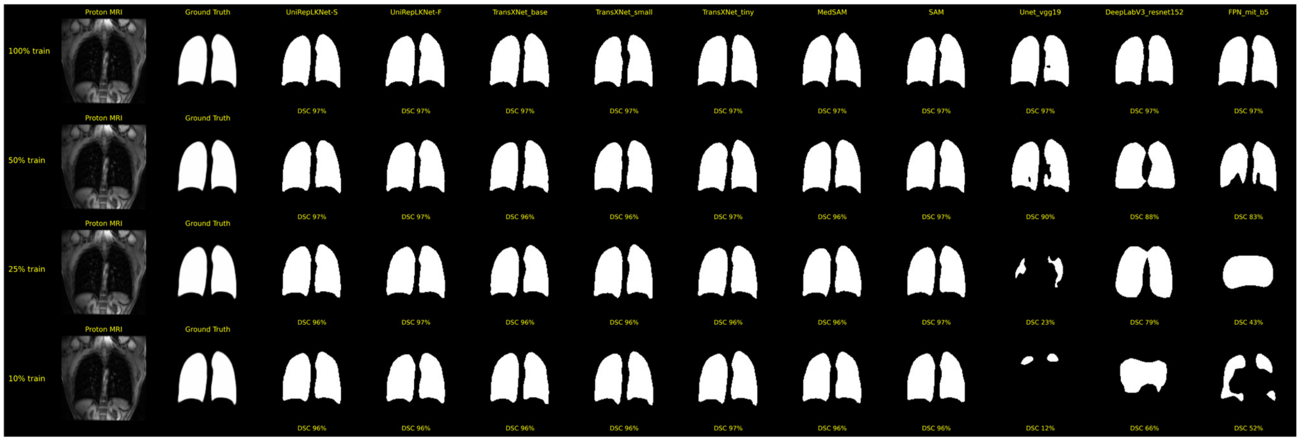

Figure S34. Qualitative comparison of proton MRI lung segmentation results across progressive data reduction scenarios and model architectures. Representative proton MRI slice (leftmost column) with corresponding ground truth segmentation (second column) and model predictions across advanced architectures (UniRepLKNet-S, UniRepLKNet-F, TransXNet variants), foundational models (MedSAM, SAM), and traditional models (UNet-VGG19, DeepLabV3-ResNet152, FPN-MIT-B5). White regions represent segmented lung areas with Dice Similarity Coefficient (DSC) values displayed above each result. Progressive data reduction from 100% to 10% training data (top to bottom rows) demonstrates the exceptional resilience of both foundational and advanced models, which consistently maintain high-quality lung boundary delineation and DSC scores above 0.94 even under extreme data constraints (10% training data). Traditional architectures exhibit progressive performance collapse, with catastrophic segmentation failures evident in the bottom two rows: UNet-VGG19 produces severely fragmented segmentations (DSC dropping to 0.12%), DeepLabV3-ResNet152 generates oversimplified oval-shaped masks (DSC: 0.66%), and FPN-MIT-B5 shows moderate but substantial degradation (DSC: 0.52%). The visual assessment clearly illustrates the statistical equivalence between foundational and advanced models while confirming their dramatic superiority over traditional approaches, providing compelling evidence that architectures incorporating large effective receptive fields are essential for robust automated lung segmentation in data-limited clinical scenarios.

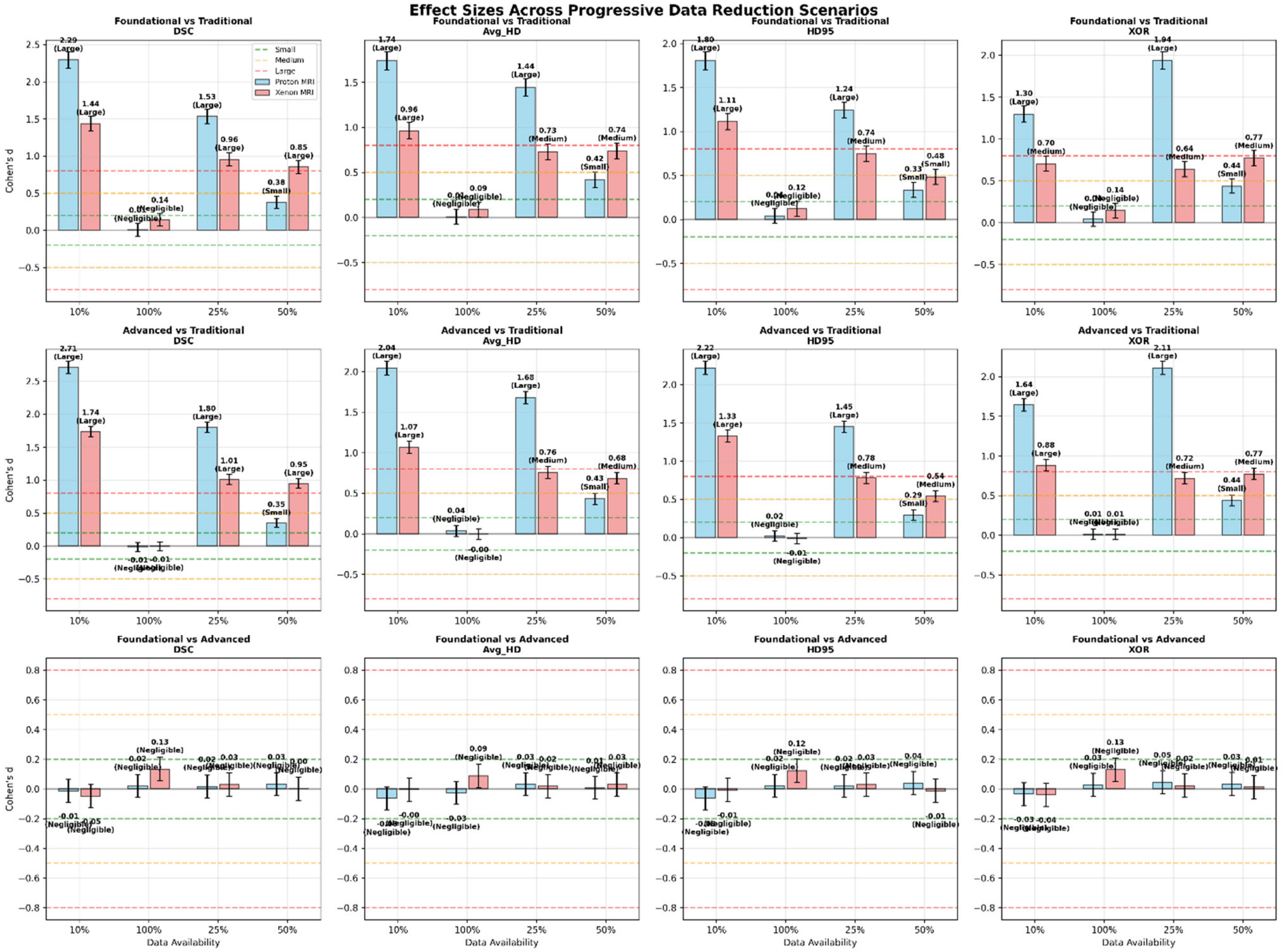

Figure S35. Cohen's d effect sizes with 95% confidence intervals comparing architectural paradigms across four evaluation metrics (DSC, Average Hausdorff Distance, 95th percentile Hausdorff Distance, and XOR error) for both proton MRI (light blue bars) and xenon MRI (light coral bars). **Top row:** Foundational vs Traditional model comparisons show negligible differences ($d < 0.2$) at 100% training data, escalating to very large effect sizes ($d > 2.0$) under extreme data scarcity (10% data). **Middle row:** Advanced vs Traditional comparisons demonstrate identical patterns with even larger effect sizes in some metrics, confirming superior data efficiency of large-kernel architectures. **Bottom row:** Foundational vs Advanced comparisons reveal negligible effect sizes ($d < 0.2$) across all data scenarios, providing statistical evidence of equivalent performance despite different underlying design principles. Dashed horizontal lines indicate conventional effect size thresholds (0.2 = small, 0.5 = medium, 0.8 = large). Error bars represent 95% confidence intervals. Effect size magnitudes and interpretations are labeled above each bar. Results demonstrate that both foundational and advanced architectures maintain statistical equivalence while significantly outperforming

traditional approaches under data constraints, with differences becoming clinically substantial (d > 0.8) at 25% training data and reaching very large magnitudes (d > 2.0) under extreme data scarcity.

Detailed Model Training Procedures and Hyperparameters

Hardware and Software Environment

Hardware Configuration:

- GPU: Two parallel NVIDIA GA102 GeForce RTX 3090 GPUs
- Framework: PyTorch with CUDA acceleration when available

Software Dependencies:

- PyTorch: Primary deep learning framework
- Segmentation Models PyTorch (smp): For traditional architectures
- Transformers (HuggingFace): For SAM/MedSAM models
- MONAI: For SAM/MedSAM preprocessing
- Albumentations: For image augmentations
- MedPy: For evaluation metrics

Data Preprocessing and Augmentation

Image Preprocessing:

- **Traditional and Advanced Models:**
    - Resize: 128×128 pixels
    - Normalization: Pixel values scaled to [0, 1]
    - Format: RGB (grayscale replicated across 3 channels)
- **Foundational Models (SAM/MedSAM):**
    - Resize: 256×256 pixels
    - Intensity scaling: [-1000, 2000] → [0, 255]
    - Spatial padding to ensure 256×256 dimensions
    - Spacing normalization: 1.5×1.5 pixel spacing
    - Format: RGB conversion from grayscale

Data Augmentation Strategy:

- Resize + **±10 degrees** rotation

Random Seeds:

- Data splitting: `random_state=32`

- Model training reproducibility: `torch.manual_seed(42)`
- DataLoader shuffling: `torch.Generator().manual_seed(42)`

Model Architectures and Configurations (Only the top performing models are reported in the main paper)

Traditional Deep Learning Models

Architectures Evaluated:

- UNet with encoders: ResNet18, ResNet34, ResNet152, VGG16, VGG19, MIT-B5
- UNet++ with encoders: ResNet18, ResNet34, ResNet152, VGG16, VGG19
- FPN with encoders: ResNet18, ResNet34, ResNet152, VGG16, VGG19, MIT-B5
- DeepLabV3 with encoders: ResNet18, ResNet34, ResNet152

Configuration:

- Input channels: 3 (RGB)
- Output classes: 1 (binary segmentation)
- Activation: None (logits output)
- Pre-trained weights: ImageNet initialization

4.2 Advanced Large-Kernel Architectures

TransXNet Variants:

- TransXNet-tiny: 12.8M parameters
- TransXNet-small: 26.9M parameters
- TransXNet-base: 48.0M parameters
- Architecture: Hybrid CNN-transformer with Dual Dynamic Token Mixer
- Input size: 128×128×3

UniRepLKNet Variants:

- UniRepLKNet-A: Depths (2,2,6,2), Dims (40,80,160,320)
- UniRepLKNet-F: Depths (2,2,6,2), Dims (48,96,192,384)
- UniRepLKNet-P: Depths (2,2,6,2), Dims (64,128,256,512)
- UniRepLKNet-N: Depths (2,2,8,2), Dims (80,160,320,640)
- UniRepLKNet-T: Depths (3,3,18,3), Dims (80,160,320,640)
- UniRepLKNet-S: Depths (3,3,27,3), Dims (96,192,384,768)
- Kernel sizes: Up to 13×13 with dilated re-parameterization
- Drop path rate: 0.1
- Layer scale initialization: 1e-6

4.3 Foundational Models

SAM Configuration:

- Base model: `facebook/sam-vit-base`
- Vision Transformer: ViT-B/16 (≈91M parameters)
- Input size: 256×256×3
- Prompt type: Bounding box (full image dimensions)
- Frozen components: Vision encoder, prompt encoder
- Trainable components: Mask decoder only

MedSAM Configuration:

- Base model: `flaviagiammarino/medsam-vit-base`
- Architecture: SAM adapted for medical imaging (≈91M parameters)
- Training procedure: Identical to SAM
- Pre-training: >1M medical image-mask pairs

Training Hyperparameters

Optimization:

- Optimizer: Adam
- Learning rate: 1e-5
- Schedule: Reduce on Plateau

Batch Configuration:

- Batch size: 16

Training Duration:

- Maximum epochs: 100
- Early stopping patience: 10 epochs
- Early stopping criterion: Validation loss/Dice improvement

Checkpoint Strategy:

- Save best model weights based on validation performance

Evaluation Metrics Implementation

- Dice Similarity Coefficient (DSC): Overlap assessment
- Hausdorff Distance 95th percentile (HD95): Boundary accuracy
- Average Hausdorff Distance (Avg HD): Overall geometric agreement
- XOR Error: Pixel-wise disagreement quantification

** Note: HD95 and AvgHD values in the main text are reported in pixels. To convert to millimetres, multiply by 3.125 (based on 3.125 mm/pixel in-plane spacing).
